# Protein phosphatase 2A regulates senescence and immunogenicity in medulloblastoma models

**DOI:** 10.1172/JCI196753

**Published:** 2026-04-23

**Authors:** Winson S. Ho, Isha Mondal, Jingjing Liu, Raymond Sun, Jiawei Huo, Chao Gao, Oishika Das, Daren Tieu, Jingqi Sun, Hanchen Lin, Peng Zhang, Jiyang Yu, Rongze Olivia Lu

**Affiliations:** 1 Department of Neurological Surgery, and; 2 Helen Diller Comprehensive Cancer Center, UCSF, San Francisco, California, USA.; 3 Department of Computational Biology, St. Jude Children’s Research Hospital, Memphis, Tennessee, USA.; 4 Department of Neurological Surgery, Malnati Brain Tumor Institute of the Robert H. Lurie Comprehensive Cancer Center, Feinberg School of Medicine, Northwestern University, Chicago, Illinois, USA.

**Keywords:** Immunology, Oncology, Cancer immunotherapy, Cellular senescence, Phosphoprotein phosphatases

## Abstract

Medulloblastoma (MB) is the most common malignant pediatric brain tumor. Current therapies are associated with substantial morbidity, and prognosis remains poor in high-risk subgroups, particularly those with *TP53* mutations or relapsed disease. Cellular senescence is a tumor-suppressive program implicated in MB, but its role in antitumor immunity remains incompletely understood. We found that protein phosphatase 2A (PP2A) regulated immunogenic senescence in MB. Genetic ablation of the PP2A catalytic subunit PP2Ac or depletion of the regulatory subunit PP2A-B56α induced senescence in MB models. PP2Ac-deficient senescent cells exhibited increased MHC class I expression and enhanced immunogenicity. In syngeneic orthotopic models, PP2Ac loss prolonged survival in an immune- and CD8^+^ T cell–dependent manner. Analysis of patient datasets showed that senescence-associated gene signatures correlated with improved survival. Single-cell transcriptomic analysis further revealed that senescent MB cells were heterogeneous and that reduced PP2A activity was associated with an immunogenic senescence state. Because the PP2A inhibitor LB-100 has limited potency and off-target effects, we developed a lipid nanoparticle (LNP) platform to deliver siRNA targeting *PPP2CA*. LNP–small-interfering PP2Ac efficiently silenced PP2Ac in vitro and, when delivered locally in vivo, prolonged survival in a CD8^+^ T cell–dependent manner. Together, these findings identify PP2A as a regulator of immunogenic senescence in MB and support PP2Ac targeting as a therapeutic strategy.

## Abstract 2

Targeting Protein Phosphatase 2A (PP2A) induces tumor cell senescence and boosts immune response in medulloblastoma, offering a potential new therapy for aggressive, treatment-resistant pediatric brain tumors.

## Introduction

Medulloblastoma (MB) is the most common pediatric brain cancer. Current standard treatments, including surgery, chemotherapy, and radiation, are associated with substantial treatment-related morbidities (1, 2). Molecular profiling has classified MB into 4 major subgroups: WNT, sonic hedgehog (SHH), group 3, and group 4 (3). Among these, group 3 MB represents the most aggressive subtype, frequently characterized by *MYC* amplification, high metastatic potential, and poor clinical outcomes (3). Despite intensive therapy, survival for high-risk and recurrent MB remains limited. In addition, MB generally shows limited responsiveness to immune checkpoint blockade targeting PD-1/PD-L1, largely due to MB’s low immunogenicity and immunosuppressive tumor microenvironment (4, 5). Therefore, identifying new therapies for these high-risk patients is imperative.

Cellular senescence is a stress-induced response that leads to irreversible cell cycle arrest (6). A transgenic model of MB tumorigenesis has suggested that senescence can limit disease progression (7). These observations suggest therapeutically inducing senescence may represent a promising strategy to suppress tumor growth. However, the role of senescence in cancer therapy remains complex and context dependent (8). Although senescence induction can suppress tumor growth, the persistence of senescent cells may promote cancer stemness, enable tumor cells to reenter the cell cycle, or contribute to immunosuppression (8–10). Conversely, recent studies have shown that senescent cancer cells can upregulate IFN signaling, thereby enhancing tumor immunogenicity and sensitizing tumors to immunotherapy (11, 12). These findings suggest senescent cells are heterogeneous, and identifying mechanisms that drive immunogenic forms of senescence may both suppress tumor-intrinsic proliferation and enhance the responsiveness of MB to immunotherapy.

Protein phosphatase 2A (PP2A) is a major serine/threonine phosphatase that counterbalances kinase activity to regulate critical signaling pathways involved in normal physiology and cancer pathobiology. PP2A functions as a heterotrimeric complex composed of a catalytic (C) subunit, a scaffolding (A) subunit, and a diverse set of regulatory (B) subunits that determine substrate specificity (13). Our group previously demonstrated that pharmacologic inhibition or genetic depletion of the PP2A catalytic subunit (PP2Ac) enhances the efficacy of immune checkpoint blockade in multiple PD-1–resistant tumor models (14–18). However, the mechanistic and potential translational role of PP2A in MB has, to our knowledge, been underexplored.

In this study, we show that PP2Ac ablation promotes an immunogenic form of senescence in MB. In both human and murine MB models, PP2Ac deletion induces robust senescence that is further augmented by radiation, an established senescence inducer and a standard therapy for MB. Through systematic screening of PP2A regulatory subunits, we identified PP2A-B56α as a key regulator that phenocopies the effects of PP2Ac deletion in promoting senescence. In syngeneic MB models, PP2Ac loss induces a robust senescence phenotype both in vitro and in vivo and elicits an adaptive immune–dependent antitumor response. Analysis of human MB datasets further revealed that senescence-associated gene signatures correlate with improved patient survival. Single-cell RNA-Seq (scRNA-Seq) analysis identified a distinct subset of immunogenic senescent tumor cells characterized by reduced PP2A activity, suggesting a critical role for PP2Ac in regulating immunogenic senescence in MB. Together, these findings identify PP2A as a key regulator of immunogenic senescence and a potential therapeutic target in MB.

Although the small-molecule PP2A inhibitor LB-100 is currently under investigation in multiple clinical trials (ClinicalTrials.gov NCT06012734, NCT03886662, and NCT06065462), its therapeutic application in CNS tumors remains limited. In a window-of-opportunity trial conducted with patients with glioblastoma, LB-100 demonstrated negligible tumor penetration (ClinicalTrials.gov NCT03027388). Furthermore, preclinical studies indicate relatively high concentrations of LB-100 are required to achieve antitumor effects (14, 19, 20), and the compound exhibits limited stability and potential off-target activity, including inhibition of *PPP5C* (21). To overcome these limitations, we developed a lipid nanoparticle–based RNA therapeutic platform for selective PP2A inhibition. Lipid nanoparticles (LNPs) are a clinically validated delivery system with demonstrated safety, manufacturing scalability, and versatility for nucleic acid therapeutics (22, 23). These LNP–small-interfering PP2Ac (LNP-siPP2Ac) particles exhibited robust cellular uptake and effective gene silencing in vitro. In vivo, local delivery of LNP-siPP2Ac in a syngeneic orthotopic MB model significantly prolonged survival and increased tumor MHC class I (MHC-I) expression, suggesting enhanced tumor immunogenicity and potential synergy with immunotherapy. Collectively, these findings establish PP2A as a critical regulator of immunogenic senescence and provide a foundation for translational strategies targeting PP2A in high-risk group 3 MB.

## Results

### PP2Ac deficiency negatively regulates proliferation and promotes NF-κB signaling.

To investigate the role of PP2Ac in MB, we first assessed its functional relevance across molecular subtypes of the disease. Because PP2A activity is largely regulated through post-translational modifications, mRNA expression levels alone are a poor proxy for enzymatic activity. We therefore applied NetBID2, a network-based algorithm that infers regulatory protein activity from transcriptomic data using gene regulatory networks (24, 25), to publicly available MB RNA-Seq datasets (26). This analysis suggested that PP2A activity is particularly prominent in groups 3 and 4 MB (Supplemental Figure 1; supplemental material available online with this article; https://doi.org/10.1172/JCI196753DS1), supporting a potential functional role for PP2A signaling in this aggressive subtype.

**Figure 1:**
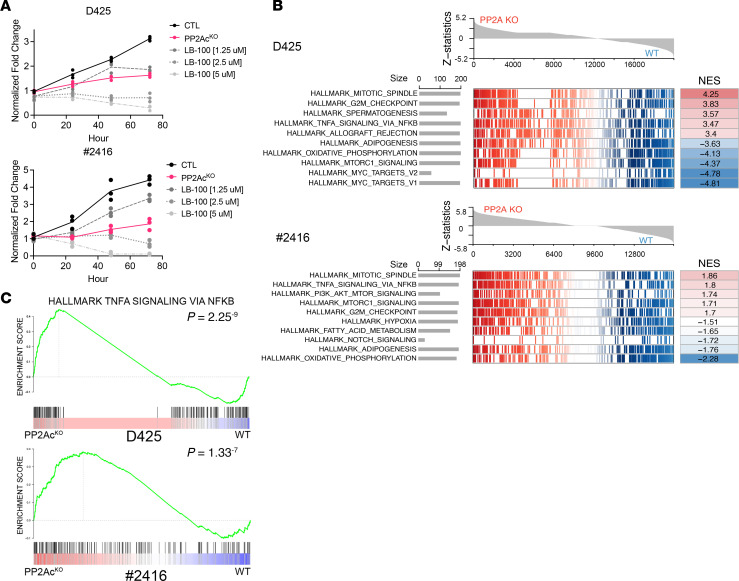
PP2A negatively regulates cell proliferation and the NF-κB signaling pathway. (**A**) Cell proliferation of D425 and #2416 MB cells measured by CCK-8 assay. WT and PP2Ac-KO cells were quantified at the indicated time points and normalized to baseline. WT cells treated with LB-100 at the indicated concentrations were analyzed in parallel. (**B**) GSEA of differentially expressed genes in PP2Ac-KO versus WT cells in D425 and #2416 lines. The top 5 upregulated (red) and downregulated (blue) hallmark pathways ranked by normalized enrichment score (NES) are shown. (**C**) Representative GSEA plots demonstrating enrichment of the Hallmark TNF-α signaling via NF-κB pathway in PP2Ac-KO compared with WT cells.

Guided by this observation, we generated genetic KO of the predominant catalytic α-isoform (*PPP2CA*) using CRISPR in murine (#2416, ref. 27; and human [D425, ATCC]) MB cell lines. Both models harbor *MYC* amplification and represent aggressive group 3 MB. KO efficiency at the protein level was confirmed by Western blot analysis (Supplemental Figure 1, A and B). We found that PP2Ac deficiency — either through genetic KO or pharmacologic inhibition using the small-molecule inhibitor LB-100 in a dose-dependent manner — resulted in a marked reduction in tumor cell proliferation in vitro compared with WT controls (Figure 1A).

To gain molecular insight into the impact of PP2Ac loss in MB, we performed RNA-Seq to obtain global gene expression profiles of WT and PP2CA-KO D425 and #2416 cells. Pathway enrichment analysis revealed several significantly altered hallmark pathways shared between both models, including mitotic spindle, G2M checkpoint, and TNF-α signaling via NF-κB (Figure 1B). Gene set enrichment analysis (GSEA) further confirmed significant enrichment of TNF-α/NF-κB signaling–related genes (Figure 1C).

Interestingly, enrichment of several proliferative signaling pathways, including mTORC1 and PI3K/AKT, occurred despite the observed reduction in cellular proliferation. This seemingly paradoxical finding is consistent with recent studies showing that PP2A inhibition can hyperactivate multiple oncogenic signaling pathways, triggering cellular stress responses that ultimately suppress tumor growth (28). Given the well-established association between NF-κB signaling and the induction of cellular senescence (9), these findings raised the possibility that PP2Ac deficiency may promote senescence in MB cells, thereby contributing to the observed reduction in proliferation.

### PP2Ac deficiency promotes cellular senescence in MB cells in vitro.

**Figure 2:**
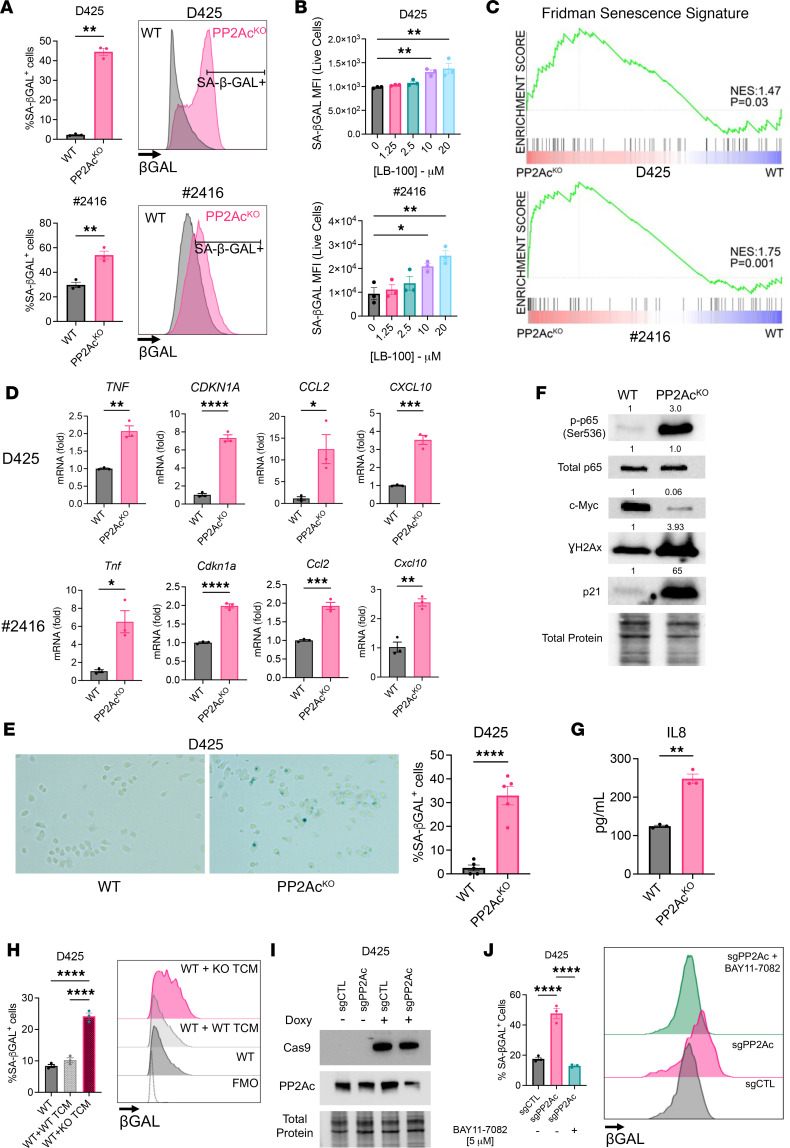
Loss of PP2Ac induces cellular senescence in MB cells. (**A**) Flow cytometric quantification of SA-β-gal activity in WT and PP2Ac-KO D425 and #2416 cells. (**B**) WT D425 and #2416 cells were treated with LB-100 for 48 hours at the indicated concentrations, followed by analysis of SA-β-gal activity by flow cytometry. (**C**) GSEA plots showing enrichment of the Fridman senescence signature in PP2Ac-KO versus WT cells in both cell lines. (**D**) RT-qPCR analysis of senescence-associated genes in WT and PP2Ac-KO D425 and #2416 cells. (**E**) Representative images and quantification of SA-β-gal staining in WT and PP2Ac-KO D425 cells assessed by light microscopy. (**F**) Immunoblot analysis of phosphorylated NF-κB (p-p65, Ser536), total NF-κB (p65), and senescence-associated proteins in WT and PP2Ac-KO D425 cells. Densitometric quantification was performed by normalizing to total protein, with WT levels set to 1. (**G**) Quantification of IL-8 protein levels in culture supernatants from WT and PP2Ac-KO D425 cells after 48 hours, measured by bead-based immunoassay. (**H**) WT D425 cells were cultured for 48 hours in CM derived from WT or PP2Ac-KO cells. SA-β-gal was subsequently measured by flow cytometry. Representative histograms are shown. (**I**) Immunoblot validation of doxycycline-inducible CRISPR-Cas9–mediated PP2Ac KO in D425 cells expressing control sgRNA (sgCTL) or PP2Ac-targeting sgRNA (sgPP2Ac). Cells were treated with doxycycline (1 μg/mL) for 3 days. (**J**) D425 cells expressing inducible Cas9 and sgPP2Ac were treated with doxycycline for 7 days, followed by the NF-κB inhibitor BAY 11-7082 or vehicle for 3 days. SA-β-gal activity was assessed on day 10 by flow cytometry. Data are shown as mean ± SEM. Statistical significance was determined using unpaired 2-tailed *t* tests, 1-way ANOVA followed by Dunnett’s or Tukey’s multiple-comparisons tests, as appropriate for each panel. Immunoblots shown are representative of 2 independent experiments. *P* < 0.05 was considered statistically significant. *P < 0.05, **P < 0.01, ***P < 0.001, ****P < 0.0001.

To test whether PP2Ac deficiency induces cellular senescence, we first examined senescence-associated β-galactosidase (SA-β-Gal) activity, a classic marker of senescence, using flow cytometry. PP2CA-KO cells exhibited a significant increase in SA-β-Gal positivity compared with WT cells (Figure 2A). Consistent with these findings, pharmacologic inhibition of PP2A using the small-molecule inhibitor LB-100 similarly increased SA-β-Gal activity in a dose-dependent manner, further supporting that PP2A inhibition promotes senescence in MB cells (Figure 2B).

To further validate this phenotype at the transcriptional level, we analyzed global gene expression changes associated with PP2Ac loss. GSEA of bulk RNA-Seq data revealed significant enrichment of a previously described senescence-associated gene signature (29) in PP2CA-KO cells in both D425 and #2416 models (Figure 2C and Supplemental Table 1). Consistent with this observation, quantitative PCR (qPCR) analysis confirmed increased expression of senescence-associated and inflammatory genes, including *CDKN1A, TNF, CCL2,* and *CXCL10*, in PP2CA-KO cells (Figure 2D). Consistent with these molecular findings, microscopy analysis revealed that PP2CA-KO D425 cells displayed the characteristic enlarged and flattened morphology associated with senescence and exhibited increased SA-β-Gal staining (Figure 2E). At the protein level, consistent with the transcriptomic findings, PP2CA-KO cells demonstrated increased phosphorylation of p65, indicating activation of NF-κB signaling (Figure 2F) as well as increased levels of γH2AX and p21, further supporting activation of a senescence program (Figure 2F).

Notably, consistent with the GSEA analysis showing downregulation of *MYC* signaling, we also observed a marked reduction in c-MYC protein levels following PP2Ac depletion (Figure 2F). Analysis of RNA-Seq datasets from PP2CA-KO D425 and #2416 cells demonstrated a modest but significant reduction in *MYC* transcript expression (Supplemental Figure 3). This observation is notable given that these are *MYC*-amplified tumors, suggesting that PP2Ac loss suppresses *MYC*-driven transcriptional programs despite oncogenic *MYC* amplification. To further support the senescence phenotype, secretion of the senescence-associated cytokine IL-8, a key component of the senescence-associated secretory phenotype (SASP), was significantly elevated in conditioned medium (CM) from PP2CA-KO cells (Figure 2G). Because SASP factors can reinforce and propagate senescence in neighboring cells, we next tested whether CM from PP2CA-KO cells could induce senescence in WT cells. Treatment of WT D425 cells with PP2CA-KO CM increased SA-β-Gal positivity compared with WT CM, indicating PP2Ac-deficient cells can promote paracrine senescence (Figure 2H).

Finally, we investigated whether NF-κB signaling mediates PP2Ac deficiency–induced senescence. We generated D425 cells expressing doxycycline-inducible Cas9 with either a nontargeting sgRNA (sgCTL) or a PP2Ac-targeting sgRNA (sgPP2Ac) (Figure 2I). Induction of PP2Ac KO markedly increased SA-β-Gal activity. To test whether this effect is mediated through NF-κB signaling, cells were treated with the NF-κB inhibitor BAY 11-7082. Immunoblot analysis confirmed effective inhibition of NF-κB signaling, as evidenced by reduced phosphorylation of p65 (Ser536) following BAY 11-7082 treatment (Supplemental Figure 4). Importantly, treatment with BAY 11-7082 significantly reduced SA-β-Gal activity in PP2Ac-deficient cells (Figure 2J). These results indicate PP2Ac loss promotes senescence in an NF-κB–dependent manner.

To determine whether this phenotype is reproducible across additional group 3 MB models, we evaluated senescence markers in the independent *MYC*-amplified MB cell line D341. Similar to D425 and #2416 cells, PP2Ac depletion in D341 cells (Supplemental Figure 2D) resulted in a significant increase in SA-β-Gal activity (Supplemental Figure 5A). Consistent with induction of a senescence program, RT-qPCR analysis demonstrated increased expression of senescence-associated and inflammatory genes, including *CDKN1A, IL8, IL6, CDKN2A*, and *CDKN2B* (Supplemental Figure 5B).

We next examined the *MYC*-amplified group 3 murine MB model CTD cell line (30) (Supplemental Figure 2E) using transcriptomic and protein-level analyses. RNA-Seq data from CTD cells revealed enrichment of senescence-associated transcriptional programs following PP2Ac depletion, including the Fridman senescence signature, as well as IFN and TNF-α/NF-κB signaling pathways (Supplemental Figure 6, A and B). Immunoblot analysis further confirmed increased expression of senescence markers p21 and γH2AX, along with increased phosphorylated NF-κB, in PP2Ac-deficient CTD cells (Supplemental Figure 6C).

### PP2Ac loss suppresses MB tumor growth and promotes senescence in vivo.

**Figure 3:**
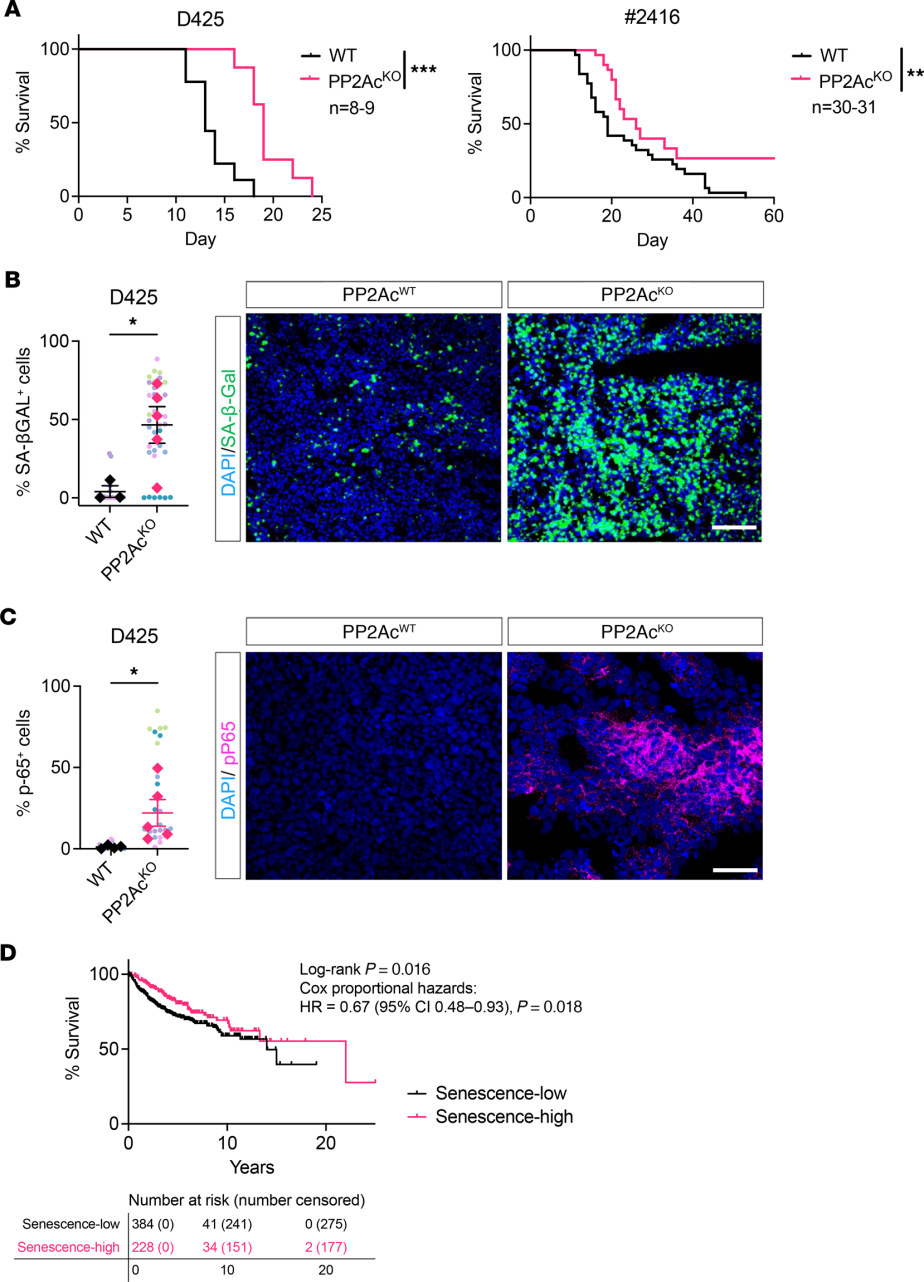
PP2Ac deficiency promotes tumor cell senescence and prolongs survival in MB models. (**A**) Kaplan-Meier survival curves of mice intracranially implanted with 3 × 10^4^ WT or PP2Ac-KO MB cells. Left: nude mice bearing D425 tumors (n = 8–9 per group). Right: C57BL/6J mice bearing #2416 tumors (n = 30–31 per group). (**B**) Representative immunofluorescence images and quantification of SA-β-gal in D425 tumors collected at endpoint. SA-β-gal (green) and nuclei (DAPI, blue). Quantification was performed from multiple ROIs per tumor. Small colored dots indicate individual ROIs nested within each biological replicate, and the larger dot indicates the mean for each tumor. Statistical analysis was performed on tumor-level means. Scale bar: 100 μm. (**C**) Representative immunofluorescence images and quantification of p-p65 in D425 tumors at endpoint. p-p65 (purple) and nuclei (DAPI, blue). Quantification and statistical analysis were conducted as in **B**. Scale bar: 100 μm. (**D**) Kaplan-Meier analysis of overall survival in patients with MB from the GSE85217 dataset stratified into senescence-high and senescence-low groups using an unbiased cutoff determined by maximally selected rank statistics (cut point = 0.1350506). All patients with available survival data were included in the analysis. Survival differences were assessed using the log-rank test. Cox proportional hazards analysis demonstrated a reduced risk of death in the senescence-high group (HR = 0.67; 95% CI 0.48–0.93). Numbers at risk are indicated below the plot. Data are shown as mean ± SEM. Statistical significance was determined using unpaired 2-tailed *t* tests or the log-rank test, as appropriate for each panel. *P* < 0.05 was considered statistically significant. *P < 0.05, **P < 0.01, ***P < 0.001.

We next examined the effect of PP2Ac loss on MB tumor growth in vivo by orthotopically implanting WT or PP2CA-KO tumor cells. Human D425 cells were implanted into immunodeficient nude mice, whereas murine #2416 cells were implanted into immunocompetent C57BL/6 mice. In both models, mice bearing PP2CA-KO tumors demonstrated significantly prolonged survival compared with those bearing WT tumors (Figure 3A), indicating that PP2Ac loss suppresses MB progression in vivo. Notably, long-term-surviving animals were observed only in the immunocompetent #2416 syngeneic model, whereas no long-term survivors were observed in the D425 xenograft model, suggesting the host immune system may contribute to durable tumor control.

To determine whether the senescence phenotype observed in vitro is also present in vivo, tumors were harvested at survival endpoint and analyzed for SA-β-Gal activity by immunofluorescence. PP2CA-KO D425 tumors had a marked increase in SA-β-Gal–positive cells compared with WT tumors (Figure 3B), confirming enhanced senescence in vivo. In the #2416 model, where tumor cells express red fluorescent protein (RFP), SA-β-Gal staining largely colocalized with RFP^+^ tumor cells, indicating that the senescence signal predominantly arises from tumor cells rather than host-derived cells (Supplemental Figure 7). Consistent with activation of NF-κB signaling observed in vitro, immunofluorescence analysis demonstrated increased phosphorylation of p65 in PP2CA-KO tumors compared with WT tumors (Figure 3C), supporting activation of NF-κB signaling in vivo.

Because the therapeutic relevance of senescence in MB remains unclear, we next examined whether senescence-associated transcriptional programs correlate with clinical outcomes. Using a publicly available transcriptomic dataset of patients with MB with available survival information from Gene Expression Omnibus (GEO) GSE85217 (3), we calculated a senescence signature score based on the Fridman senescence gene set (29). We first evaluated the senescence score as a continuous variable in a Cox proportional hazards model, which showed a consistent but nonstatistically significant association with survival (HR = 0.35; 95% CI 0.10–1.20; *P* = 0.092) (Supplemental Figure 8A). We next used maximally selected rank statistics to identify an unbiased cutoff for stratifying tumors into senescence-high and senescence-low groups. Kaplan-Meier analysis based on this cutoff showed that patients with senescence-high tumors had significantly improved overall survival compared with those with senescence-low tumors (log-rank *P* = 0.016) (Figure 3D). Consistently, Cox proportional hazards analysis of this categorical grouping showed a significantly reduced risk of death in the senescence-high group (HR = 0.67; 95% CI 0.48–0.93; *P* = 0.018) (Supplemental Figure 8B), suggesting activation of senescence-associated programs may be associated with favorable clinical outcomes in MB.

### PP2Ac deficiency enhances radiation-induced senescence in MB cells.

**Figure 4:**
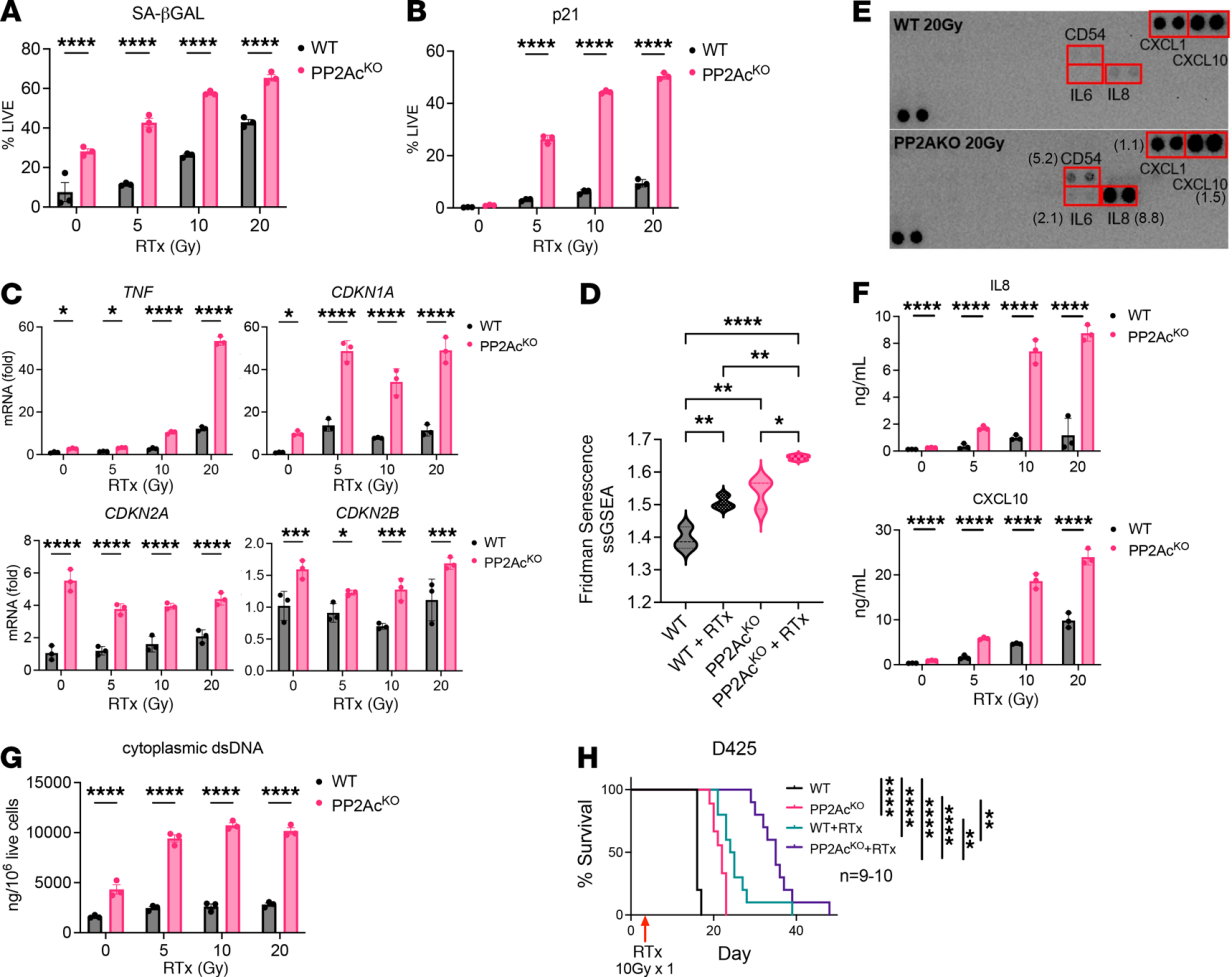
PP2Ac deficiency enhances radiation-induced senescence and immune-stimulatory cytokine expression in MB cells. (**A**–**C**) D425 WT and PP2Ac-KO cells were treated with RTx, and senescence markers were assessed over time. (**A**) SA-β-gal activity and (**B**) p21 expression quantified by flow cytometry. (**C**) RT-qPCR analysis of senescence- and SASP-associated genes including *TNF, CDKN1A, CDKN2A*, and *CDKN2B*. (**D**) Fridman senescence signature scores calculated by ssGSEA from RNA-Seq of WT and PP2Ac-KO cells with or without RTx (10 Gy). (**E**) CM collected 24 hours after irradiation (10 Gy) was analyzed using the Human Cytokine Array Proteome Profiler. (**F**) Cytokine levels (CXCL10 and IL-8) in culture supernatants measured by bead-based immunoassay following RTx. (**G**) Quantification of cytoplasmic dsDNA in WT and PP2Ac-KO cells 48 hours after irradiation. (**H**) Kaplan-Meier survival curves of nude mice implanted intracranially with WT or PP2Ac-KO D425 cells followed by focal brain irradiation (10 Gy ×1). Data are shown as mean ± SEM. Statistical significance was determined using unpaired 2-tailed *t* tests, 1-way ANOVA followed by Tukey’s multiple-comparisons test, or the log-rank test, as appropriate for each panel. *P <* 0.05 was considered statistically significant. *P < 0.05, **P < 0.01, ***P < 0.001, ****P < 0.0001.

Radiation therapy (RTx) is a well-established inducer of cellular senescence (31) and remains a cornerstone of MB treatment. To investigate whether PP2Ac loss modulates RTx-induced senescence, D425 WT and PP2Ac-KO cells were exposed to increasing doses of RTx and analyzed 48 hours later. Flow cytometry demonstrated that PP2Ac-KO cells had significantly higher SA-β-Gal positivity compared with WT cells across all RTx doses tested (Figure 4A). Consistently, PP2Ac-KO cells had increased p21 expression after RTx relative to WT cells (Figure 4B). RTx also induced senescence markers in WT cells, and this RTx-alone effect was shown explicitly in an independent analysis comparing within-genotype RTx responses (Supplemental Figure 9A).

To further define senescence-associated transcriptional responses, we quantified senescence- and inflammatory genes by RT-qPCR. PP2Ac-KO cells had significantly increased expression of *TNF*, *CDKN1A, CDKN2A*, and *CDKN2B* across RTx doses compared with WT cells (Figure 4C). To evaluate global senescence programs, we performed RNA-Seq and calculated the Fridman senescence signature by single-sample GSEA (ssGSEA). The combination of PP2Ac loss and RTx produced the highest senescence signature scores relative to either perturbation alone (Figure 4D).

Because senescent cells can reshape the tumor microenvironment through the SASP, we next profiled secreted cytokines after RTx. Conditioned media collected 24 hours after RTx (20 Gy) were analyzed using a cytokine array, which revealed increased secretion of inflammatory/SASP factors in PP2Ac-KO cells, including IL-6, CD54, IL-8, and CXCL10 (Figure 4E). We focused on IL-8 and CXCL10 for quantitative validation because IL-8 is a canonical NF-κB–dependent SASP cytokine, and CXCL10 is an IFN-stimulated chemokine implicated in recruitment of cytotoxic T cells and antitumor immune responses. Bead-based immunoassays confirmed significantly increased secretion of IL-8 and CXCL10 in PP2Ac-KO cells compared with WT cells across RTx doses (Figure 4F). RTx-alone induction of these cytokines in WT cells, and the within-genotype RTx effect, are further shown in Supplemental Figure 9B.

Previously, we reported that PP2Ac loss impairs DNA damage repair and promotes accumulation of cytoplasmic DNA (14). Consistent with this observation, PP2Ac-KO cells exhibited significantly increased levels of cytoplasmic dsDNA across RTx doses compared with WT cells (Figure 4G), supporting enhanced activation of DNA damage–associated inflammatory signaling pathways.

Finally, to test whether these findings translate in vivo, WT or PP2Ac-KO D425 cells were orthotopically implanted into the cerebellum of nude mice followed by cranial RTx (10 Gy × 1). RTx-treated mice bearing PP2Ac-KO tumors exhibited significantly prolonged survival compared with all other groups (Figure 4H), indicating that PP2Ac loss enhances RTx efficacy in vivo. In addition, a fractionated RTx regimen (2 Gy × 3) showed a similar trend (Supplemental Figure 9C), supporting that the phenotype is not restricted to a single RTx schedule.

### The regulatory PP2A subunit PPP2R5A (B56α) suppresses senescence in MB cells.

**Figure 5:**
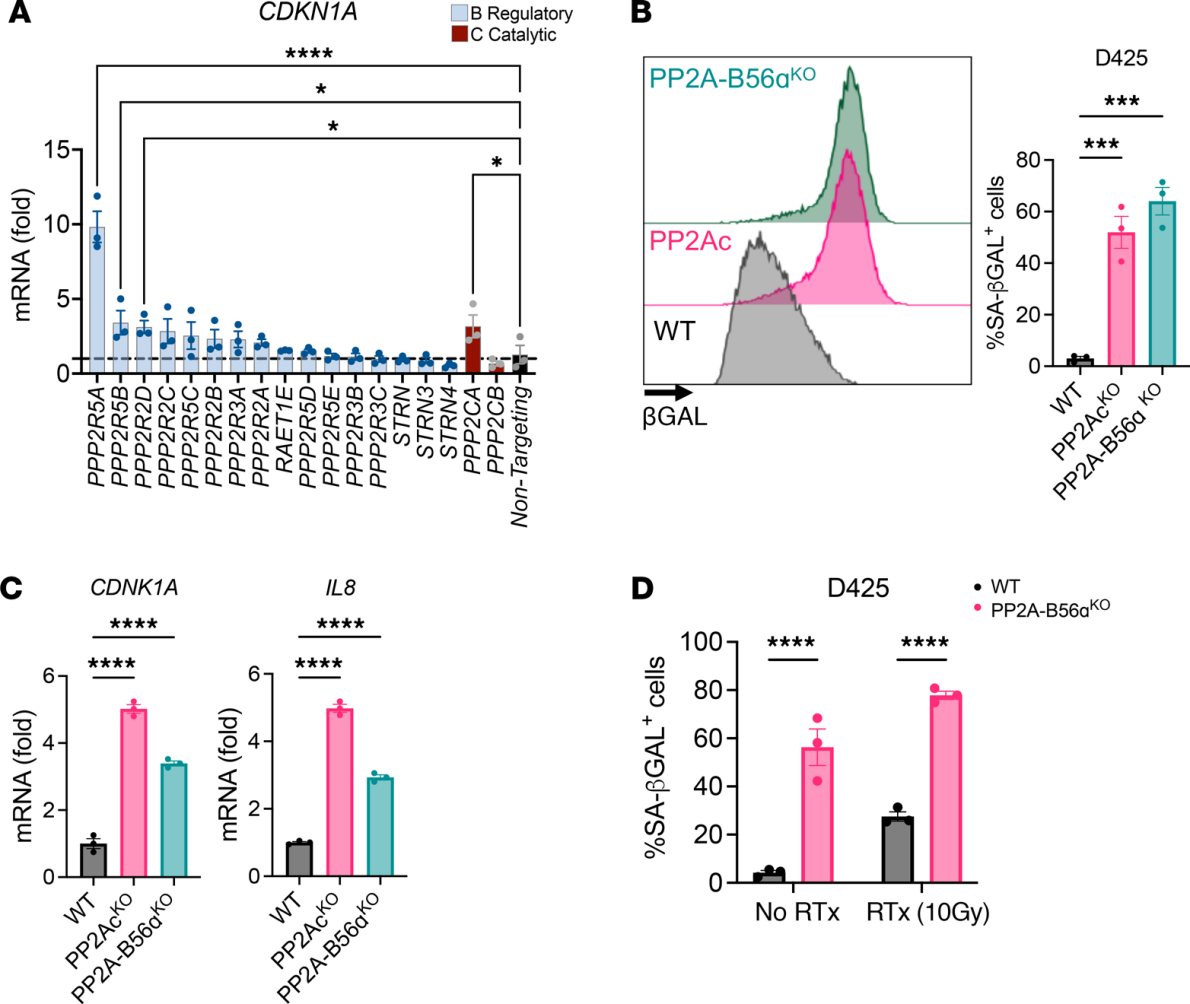
Regulatory subunit *PPP2R5A* (PP2A-B56α) negatively regulates senescence in MB cells. (**A**) siRNA screen targeting 20 PP2A subunits (*n* = 16 regulatory and 2 catalytic) in D425 cells. At 48 hours after transfection, *CDKN1A* (p21) expression was measured by RT-qPCR and normalized to a nontargeting siRNA control. PP2A subunits are grouped according to structural class. (**B**) Flow cytometric quantification of SA–β-gal in WT and PP2A-B56α–KO (*PPP2R5A*-KO) D425 cells. Representative histograms and quantification of the percentage of SA–β-gal–positive cells are shown. (**C**) RT-qPCR analysis of *CDKN1A* and *IL8* expression in WT and PP2A-B56α–KO cells. (**D**) WT and PP2A-B56α–KO D425 cells were treated with RTx (10 Gy), and SA–β-gal activity was measured by flow cytometry 48 hours after irradiation. Data are shown as mean ± SEM. Statistical significance was determined using unpaired 2-tailed *t* tests or 1-way ANOVA followed by Dunnett’s multiple-comparisons test, as appropriate for each panel. *P <* 0.05 was considered statistically significant. ***P < 0.001, ****P < 0.0001.

The PP2A holoenzyme is composed of a scaffolding A subunit, a catalytic C subunit, and a regulatory B subunit that determines substrate specificity. Regulatory B subunits belong to 4 structurally distinct families (B55, B56, PR70/72, and STRN) that share little sequence similarity but confer diverse functional specificity to the PP2A complex (32). To identify which PP2A regulatory subunit modulates senescence in MB cells, we performed a loss-of-function siRNA screen targeting 20 PP2A subunits, including regulatory B subunits as well as the catalytic and scaffolding subunits, in D425 cells. Senescence induction was assessed by measuring expression of the senescence marker *CDKN1A* (p21) 48 hours after transfection. Among all PP2A subunits tested, knockdown of *PPP2R5A*, which encodes the regulatory subunit PP2A-B56α, produced the most pronounced increase in *CDKN1A* expression (Figure 5A), suggesting that PP2A-B56α negatively regulates senescence signaling in MB cells. To validate this finding, we generated *PPP2R5A* KO (PP2A-B56αKO) D425 cells using CRISPR/Cas9 (Supplemental Figure 1C). Consistent with the screening results, PP2A-B56αKO cells displayed a marked increase in SA-β-Gal–positive cells, comparable to that observed with PP2Ac deficiency (Figure 5B). At the transcriptional level, PP2A-B56αKO cells also showed increased expression of *CDKN1A* and the SASP cytokine *IL8* (Figure 5C), supporting activation of a senescence program.

We next examined whether loss of PP2A-B56α also modulates radiation-induced senescence. Similar to PP2Ac deficiency, PP2A-B56αKO cells exhibited significantly increased SA-β-Gal activity after RTx (10 Gy) compared with WT cells (Figure 5D). These findings indicate the PP2A-B56α regulatory subunit functions as a key negative regulator of senescence signaling in MB cells, phenocopying the effects observed with catalytic PP2Ac loss.

### PP2Ac deficiency promotes IFN signaling and immunogenic senescence in MB.

**Figure 6:**
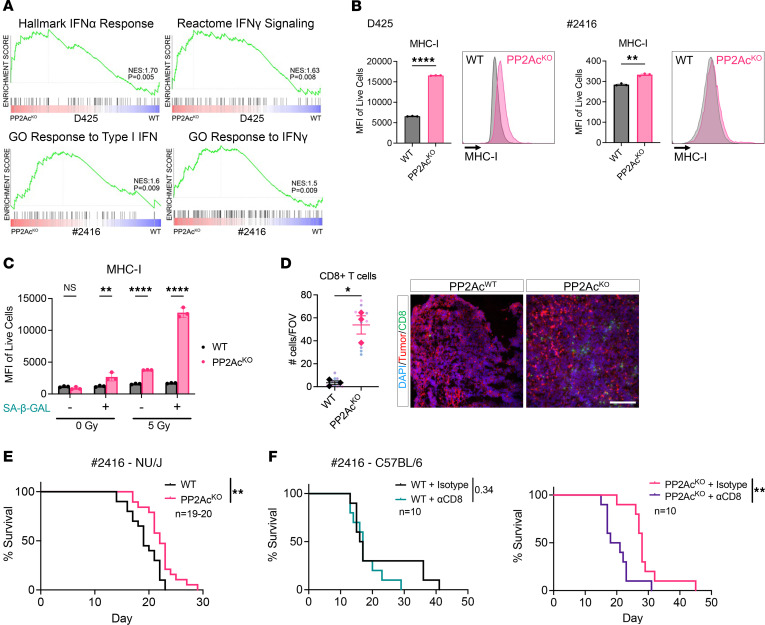
PP2Ac deficiency enhances immunogenic senescence in MB tumors. (**A**) GSEA plots demonstrating enrichment of type I and type II IFN response signatures in PP2Ac-KO versus WT D425 and #2416 cells. (**B**) Flow cytometric quantification of MHC-I surface expression in WT and PP2Ac-KO D425 and #2416 cells. Representative histograms and quantification of MFI are shown. (**C**) D425 cells were treated with 5 Gy RTx, and MHC-I expression was measured 48 hours later in SA-β-gal–positive and SA-β-gal–negative populations by flow cytometry. (**D**) Immunofluorescence analysis of CD8^+^ T cell infiltration in #2416 tumors. CD8 (green), tumor cells (RFP, red), and nuclei (DAPI, blue). Quantification was performed from multiple ROIs per tumor. Small colored dots represent individual ROIs nested within each biological replicate, and the larger dot represents the mean value for each tumor. Statistical analysis was performed using tumor-level means. Scale bar: 20 μm. (**E**) Kaplan-Meier survival analysis of nude mice bearing intracranial #2416 tumors derived from WT or PP2Ac-KO cells. (**F**) Kaplan-Meier survival analysis of C57BL/6J mice bearing intracranial #2416 tumors treated with anti-CD8 antibody (clone 2.43) or isotype control. Treatment was initiated on day 3 after implantation (250 μg/mouse i.p., twice weekly). Data are shown as mean ± SEM. Statistical significance was determined using unpaired 2-tailed *t* tests or the log-rank test, as appropriate for each panel. *P <* 0.05 was considered statistically significant. *P < 0.05, **P < 0.01, ****P < 0.0001. FOV, field of view; GO, Gene Ontology; NES, normalized enrichment score.

Recent studies suggest senescent tumor cells can enhance tumor immunogenicity through activation of type I and type II IFN signaling pathways (11, 12). Building on our prior observation that PP2Ac deficiency increases secretion of the IFN-stimulated chemokine CXCL10, we hypothesized that PP2Ac loss promotes immunogenic senescence, characterized by activation of IFN signaling and increased tumor immunogenicity. To test this, we performed GSEA comparing the transcriptional profiles of PP2Ac-KO and WT MB cells. We observed significant enrichment of type I and type II IFN response pathways in both D425 and #2416 PP2Ac-KO cells (Figure 6A). Because IFN signaling enhances antigen presentation, we next examined the expression of MHC-I, a key molecule required for CD8^+^ T cell–mediated tumor recognition. Flow cytometry analysis demonstrated significantly increased MHC-I surface expression in PP2Ac-KO D425 and #2416 cells compared with WT cells (Figure 6B).

To determine whether increased antigen presentation is linked to senescence induction, we stratified D425 cells into senescent (SA-β-Gal^+^) and nonsenescent (SA-β-Gal^–^) populations following sublethal irradiation (5 Gy), a dose selected to induce senescence while preserving high overall cell viability. We then quantified MHC-I expression and found that SA-β-Gal^+^ cells exhibited markedly higher MHC-I levels than do SA-β-Gal^–^ cells, with the strongest induction observed in PP2Ac-KO senescent cells (Figure 6C). In contrast, MHC-I expression in nonsenescent cells was only modestly affected. These findings indicate the increase in antigen presentation associated with PP2Ac deficiency is primarily linked to the senescent tumor cell population, suggesting PP2Ac loss programs senescence toward an immunogenic phenotype.

We next examined whether these changes translate into enhanced antitumor immune responses in vivo. Immunofluorescence analysis of syngeneic #2416 MB tumors demonstrated a significant increase in tumor-infiltrating CD8^+^ T cells in PP2Ac-KO tumors compared with WT tumors (Figure 6D). In our previous in vivo studies, PP2Ac-KO tumors demonstrated improved survival in both xenograft (D425) and syngeneic (#2416) MB models. However, durable long-term survival was observed only in the immunocompetent #2416 model, suggesting immune-mediated mechanisms may contribute to tumor control. To further evaluate the contribution of the immune system, we implanted #2416 tumors into immunodeficient nude mice, which lack functional T cells. Although PP2Ac-KO tumors still exhibited a modest but significant survival advantage compared with WT tumors (Figure 6E), the magnitude of this benefit was reduced relative to that observed in immunocompetent hosts. These findings indicate PP2Ac deficiency exerts both tumor-intrinsic growth suppression and immune-mediated antitumor effects in vivo. To directly test whether adaptive immunity contributes to the survival benefit, we implanted #2416 tumors into immunocompetent C57BL/6 mice and depleted CD8^+^ T cells using anti-CD8 antibodies. CD8^+^ T cell depletion significantly reduced the survival advantage conferred by PP2Ac deficiency (Figure 6F). Collectively, these findings demonstrate that although PP2Ac loss induces tumor-intrinsic senescence, optimal tumor control requires CD8^+^ T cell–mediated antitumor immunity, supporting a model in which PP2Ac deficiency promotes immunogenic senescence that enhances tumor antigen presentation and stimulates antitumor immune responses.

### Low PP2A activity is associated with immunogenic senescence in human MB.

**Figure 7:**
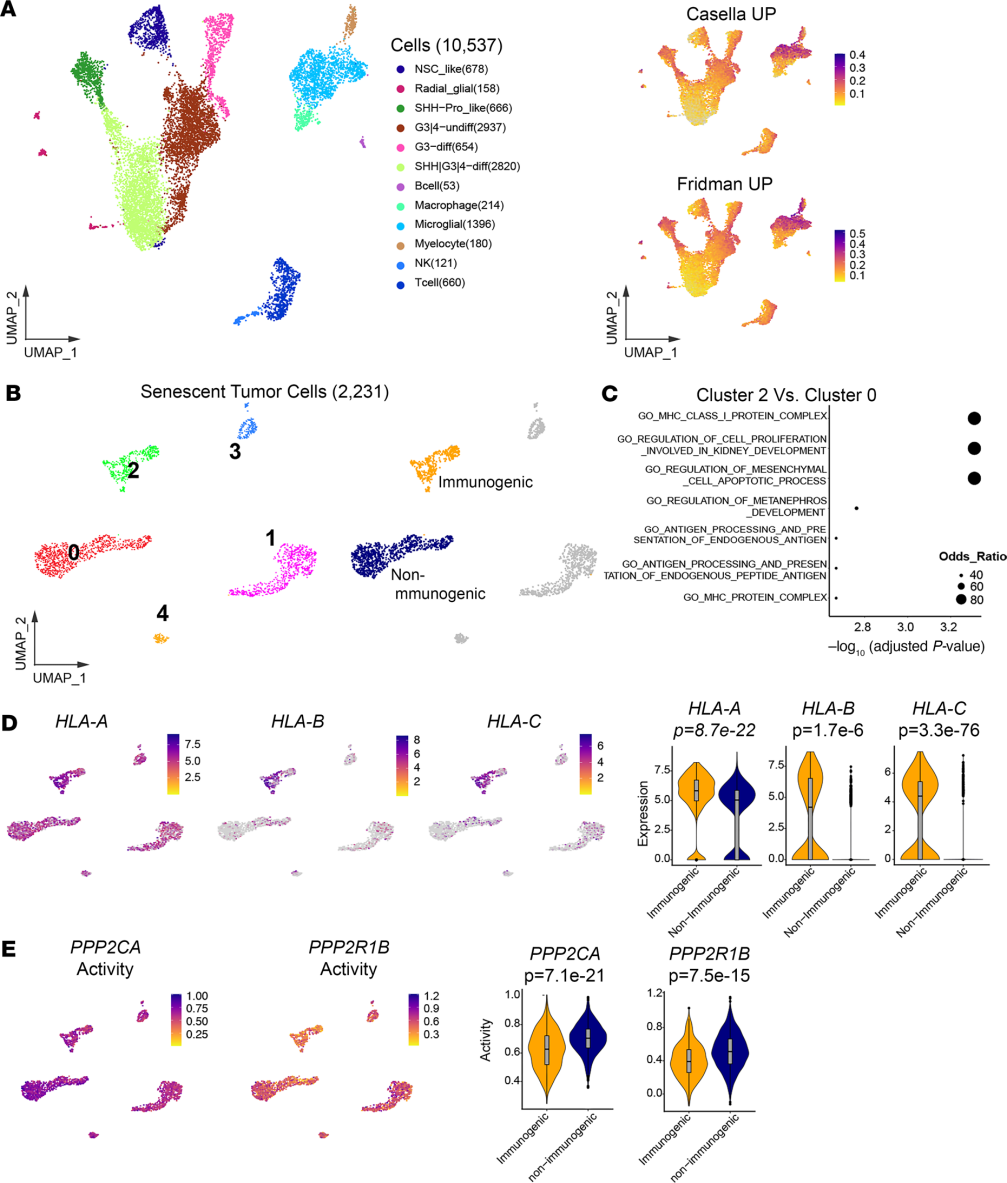
Low *PPP2CA* and *PPP2R1B* activity is associated with an immunogenic senescence phenotype in human MB. Publicly available scRNA-Seq data from 28 MB tumors were analyzed. (**A**) Uniform Manifold Approximation and Projection (UMAP) visualization of 10,537 tumor cells annotated by cell type based on the original study. Senescence scores for malignant cells were calculated using 2 published gene signatures (Casella et al., ref. 34; and Fridman and Tainsky, ref. 29) and displayed as UMAP feature plots. (**B**) Identification of 2,231 tumor cells with high senescence scores. Within group 3/4 MB malignant cells, senescent clusters were classified as immunogenic (cluster 2) or nonimmunogenic (cluster 0). (**C**) Gene Ontology (GO) enrichment analysis of differentially expressed genes between immunogenic and nonimmunogenic senescent clusters, highlighting pathways related to MHC-I complex formation and antigen presentation. Enrichment was assessed using Fisher’s exact test with FDR correction; odds ratios are shown. (**D**) UMAP feature plots and violin plots showing increased expression of MHC-I genes (*HLA-A, HLA-B, HLA-C*) in immunogenic senescent clusters. *P* values were calculated using 2-sided Wilcoxon rank-sum tests. (**E**) NetBID2-inferred activity of *PPP2CA* (catalytic subunit) and *PPP2R1B* (scaffold subunit) in immunogenic versus nonimmunogenic senescent clusters. UMAPs display inferred activity scores, with corresponding violin plots summarizing activity distributions. Adjusted *P* values were calculated using 2-sided Wilcoxon rank-sum tests.

To assess the relevance of our findings on PP2A-mediated immunogenic senescence in human MB, we analyzed a publicly available scRNA-Seq dataset (GEO GSE155446) comprising 28 MB tumors (33). Applying 2 published senescence gene signatures (29, 34) to 10,537 malignant cells identified 2,231 tumor cells with high senescence scores (Figure 7A and Supplemental Table 1). These senescent tumor cells were subsequently re-clustered, revealing 5 distinct senescent subpopulations, indicating substantial heterogeneity among senescent tumor states in MB (Figure 7B). Consistent with known molecular subgroup differences, SHH tumors segregated clearly from group 3 and group 4 (G3/4) tumors (Supplemental Figure 10, A and B). Consistent with our experimental focus on group 3 MB, we concentrated our analysis on malignant cells within the group 3/4 population, where sufficient numbers of cells were available for clustering analysis. We identified 2 major senescent subclusters (clusters 0 and 2). Gene Ontology enrichment analysis of genes upregulated in cluster 2 versus cluster 0 revealed significant enrichment for pathways related to MHC-I protein complex formation and antigen presentation (Figure 7C). Type I and type II IFN response signatures were also significantly enriched (Supplemental Data 1). Based on these features, we designated cluster 2 as an “immunogenic senescent” population, whereas cluster 0, which showed enrichment of epithelial–mesenchymal transition programs (Supplemental Figure 10C), was classified as “nonimmunogenic senescent” tumor cells. Consistent with enhanced antigen presentation in the immunogenic senescent cluster, expression of MHC-I genes (*HLA-A, HLA-B*, and *HLA-C*) was significantly increased in cluster 2 compared with cluster 0 (Figure 7D).

To investigate molecular drivers associated with this immunogenic senescence phenotype, we applied NetBID2, a network-based activity inference framework that integrates regulatory network information to infer protein activity rather than transcript abundance (24, 25). Notably, the inferred activity of the PP2A catalytic subunit (*PPP2CA*) and scaffolding subunit (*PPP2R1B*) was significantly lower in immunogenic senescent tumor cells compared with nonimmunogenic senescent cells (Figure 7E), whereas their RNA expression levels were not significantly different. These findings suggest that reduced PP2A activity is associated with an immunogenic senescence state in human MB, consistent with our experimental observations that PP2Ac loss promotes senescence and enhances tumor immunogenicity. Together, these human single-cell data support our experimental findings and suggest reduced PP2A activity may represent a conserved mechanism linking senescence and tumor immunogenicity in MB.

### Local delivery of LNP-siPP2Ac promotes senescence and tumor immunogenicity in MB.

Our findings indicate inhibition of PP2A activity induces senescence and enhances tumor immunogenicity in aggressive MB models. Consistent with this, pharmacologic inhibition using the small-molecule PP2A inhibitor LB-100 reduced tumor cell proliferation and induced senescence phenotypes in MB cells (Figure 1A and Figure 2B). Notably, these effects required mid- to high micromolar concentrations of LB-100 to phenocopy PP2Ac genetic depletion, suggesting potential potency limitations of pharmacologic PP2A inhibition. Although LB-100 is currently being evaluated in several clinical trials for non-CNS malignancies (ClinicalTrials.gov NCT06012734, NCT03886662, and NCT06065462), a window-of-opportunity trial in recurrent glioblastoma demonstrated negligible penetration of LB-100 into brain tumor tissue (ClinicalTrials.gov NCT03027388). In addition, LB-100 can inhibit other serine/threonine phosphatases, including PP5, indicating incomplete specificity for PP2A (21). Together, these delivery, potency, and specificity limitations may restrict the therapeutic potential of small-molecule PP2A inhibitors for CNS tumors.

**Figure 8:**
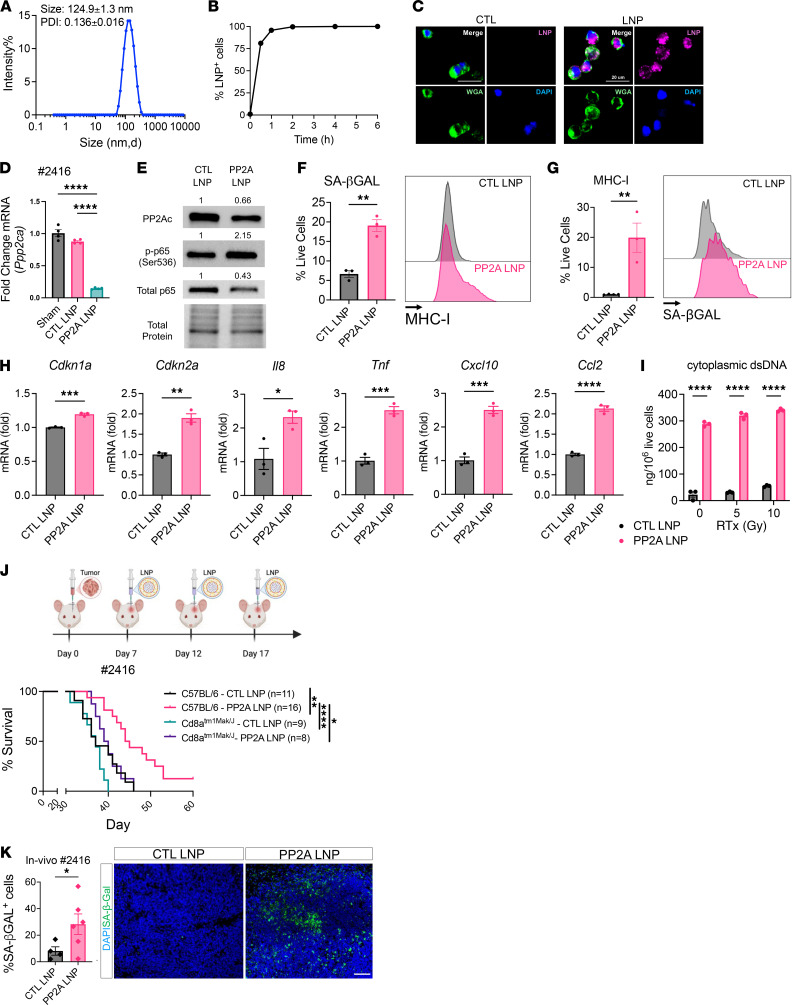
Local delivery of LNP-siPP2Ac promotes tumor immunogenicity in MB. (**A**) (A) Dynamic light scattering analysis of PP2A LNP. Mean particle diameter was 124.9 ± 1.3 nm with a polydispersity index (PDI) of 0.136 ± 0.016. (**B**) Time-course analysis of fluorescently labeled LNP uptake in #2416 cells quantified by flow cytometry. (**C**) Immunofluorescence images of #2416 cells treated with fluorescently labeled PP2A LNP or vehicle control. Cells were stained with wheat germ agglutinin (WGA) and DAPI. (**D**) RT-qPCR analysis of Ppp2ca (PP2Ac) mRNA in #2416 cells treated with CTL LNP or PP2A LNP. (**E**) Immunoblot analysis of PP2Ac, phospho-p65 (Ser536), and total p65 in #2416 cells treated with CTL LNP or PP2A LNP for 72 hours. Total protein staining served as a loading control. Densitometric values were normalized to total protein and expressed relative to CTL LNP. (**F** and **G**) SA-β-gal activity (F) and MHC-I surface expression (**G**) in #2416 cells treated with CTL LNP or PP2A LNP for 72 hours. (**H**) RT-qPCR analysis of senescence- and SASP-associated genes in #2416 cells 72 hours after treatment with CTL LNP or PP2A LNP. (**I**) Cytoplasmic dsDNA in #2416 cells treated with CTL LNP or PP2A LNP, followed by irradiation (RTx) at the indicated doses. (**J**) Schematic of the intratumoral delivery schedule and Kaplan-Meier survival analysis. C57BL/6 or CD8-deficient (Cd8a^tm1Mak/J) mice bearing orthotopic #2416 tumors received 3 μL CTL LNP or PP2A LNP on days 7, 10, and 13 after implantation (*n* = 8–16/group; combined results from 2 independent experiments). (**K**) Representative immunofluorescence images and quantification of SA-β-gal in #2416 tumors harvested at endpoint. SA-β-gal (green) and nuclei (DAPI, blue). Scale bar: 100 μm. Data are shown as mean ± SEM. Statistical significance was determined using unpaired 2-tailed *t* tests, 1-way ANOVA followed by Tukey’s multiple-comparisons test, or the log-rank test, as appropriate. *P < 0.05, **P < 0.01, ***P < 0.001, ****P < 0.0001.

To overcome these challenges, we developed an LNP formulation encapsulating siRNA targeting PP2Ac (PP2A-LNP) along with a nontargeting control nanoparticle (CTL LNP) to enable efficient and specific local silencing of PP2Ac within brain tumors. Dynamic light scattering analysis demonstrated that the nanoparticles were uniform in size (~125 nm) with a low polydispersity index, indicating a well-defined nanoparticle formulation (Figure 8A). Flow cytometric analysis revealed rapid cellular uptake of fluorescently labeled LNPs in #2416 MB cells, with the majority of cells becoming LNP-positive within 1 hour of exposure (Figure 8B). Confocal microscopy further confirmed efficient intracellular accumulation of LNPs in treated cells (Figure 8C). Consistent with this efficient uptake, treatment with PP2A-LNP resulted in robust suppression of *PPP2CA* mRNA expression compared with both sham-treated cells and CTL LNP–treated cells (Figure 8D). Immunoblot analysis further confirmed effective knockdown of PP2Ac and demonstrated increased phosphorylation of NF-κB (p-p65, Ser536) following PP2A-LNP treatment, consistent with activation of NF-κB signaling downstream of PP2Ac suppression (Figure 8E).

Functionally, PP2A-LNP treatment recapitulated the senescence phenotype observed with genetic PP2Ac loss. PP2A-LNP significantly increased SA-β-gal activity in #2416 cells compared with CTL LNP (Figure 8F) and also increased MHC-I surface expression, consistent with enhanced tumor immunogenicity (Figure 8G). At the transcriptional level, PP2A-LNP treatment induced expression of senescence- and SASP-associated genes, including *CDKN1A, CDKN2A, IL8, TNF, CXCL10*, and *CCL2* (Figure 8H). In addition, PP2Ac silencing increased the accumulation of cytoplasmic dsDNA with or without irradiation (Figure 8I), consistent with enhanced activation of DNA damage–associated inflammatory signaling pathways.

To evaluate therapeutic efficacy in vivo, we orthotopically implanted #2416 tumors into mice and delivered PP2A-LNP or CTL LNP locally through intracranially implanted cannulas according to the treatment schedule shown in Figure 8J. In immunocompetent C57BL/6 mice, local administration of PP2A-LNP significantly prolonged survival compared with CTL LNP treatment. Importantly, the survival benefit was largely abolished in CD8-deficient mice, indicating that the therapeutic effect of PP2Ac silencing depends on CD8^+^ T cell–mediated antitumor immunity (Figure 8J).

Consistent with induction of tumor senescence in vivo, tumors harvested at the survival endpoint from mice treated with PP2A-LNP had a significantly higher proportion of SA-β-gal–positive tumor cells compared with CTL LNP–treated tumors (Figure 8K). Together, these findings demonstrate PP2A-LNP–mediated PP2Ac silencing induces tumor senescence, enhances tumor immunogenicity and promotes CD8^+^ T cell–dependent antitumor responses, supporting the translational potential of PP2Ac-targeted nanotherapy as an immunomodulatory strategy for treating MB.

## Discussion

This study identifies PP2Ac, together with the regulatory subunit PP2A-B56α, as a key regulator of immunogenic senescence in MB. We demonstrate that genetic ablation of PP2Ac induces robust senescence in MB cells, resulting in reduced proliferation in vitro and prolonged survival in vivo in an adaptive immunity–dependent manner. PP2Ac deficiency also synergizes with RTx to enhance senescence and improve therapeutic efficacy. Importantly, PP2Ac loss drives a shift toward an immunogenic senescence phenotype characterized by increased antigen presentation, IFN signaling, and enhanced CD8^+^ T cell infiltration. Consistent with these findings, scRNA-Seq analysis of human MB tumors reveals that senescent tumor cells are heterogeneous and that reduced PP2A activity is associated with an immunogenic senescent state. Finally, we show that local delivery of PP2Ac-targeting siRNA using LNP-siPP2Ac enhances tumor immunogenicity and improves survival in vivo. Together, these findings establish PP2A as a central regulator of immunogenic senescence and highlight PP2Ac as a potential therapeutic target in MB.

Cellular senescence is a well-recognized tumor-suppressive mechanism that typically depends on the p53 and p16 pathways to enforce irreversible cell-cycle arrest in response to cellular stress (9). In a genetically engineered model of MB, senescence has been shown to limit tumor progression, supporting its relevance in MB biology (7). Our findings demonstrate that PP2Ac loss induces senescence through a p53-independent mechanism, revealing what we believe to be an unrecognized role for PP2A in regulating tumor cell fate. This observation is particularly relevant in aggressive MB subgroups, where *TP53* mutations frequently arise in relapsed disease (35) and are associated with poor clinical outcomes (35, 36). These findings suggest PP2A inhibition may provide a strategy to therapeutically induce senescence even in tumors with defective p53 signaling. Although senescence suppresses tumor cell proliferation, its role within the tumor microenvironment is complex (8). Persistent senescent cells may promote tumor recurrence or cellular plasticity under certain conditions (9, 10). However, emerging studies indicate senescent cancer cells can also stimulate antitumor immune responses through IFN signaling and the SASP (11, 12). Our findings support this latter model in MB. We demonstrate that tumors with higher senescence signatures are associated with improved survival in human MB datasets. Moreover, single-cell transcriptomic analysis reveals that senescent MB cells exist in heterogeneous states, including immunogenic and nonimmunogenic subpopulations. Computational inference of regulatory activity further identified reduced PP2A activity in immunogenic senescent clusters, linking PP2A inhibition to a senescence program that enhances tumor immunogenicity.

PP2A has often been considered a tumor suppressor (37), and several groups have pursued therapeutic strategies aimed at reactivating specific tumor-suppressive PP2A holoenzymes. In particular, small-molecule activators of PP2A have been developed to stabilize defined PP2A complexes and restore phosphatase activity toward oncogenic kinase pathways (38–41). These studies demonstrate that activation of selected PP2A holoenzymes can suppress tumor growth across multiple cancer models. In contrast, our study examines a complementary concept: that partial or non–holoenzyme-selective attenuation of PP2A activity may produce net antitumor benefit through pleiotropic mechanisms (17–20, 42, 43). Recently, we and others have shown that PP2A deficiency, either in tumor cells (14) or tumor-associated myeloid cells (15), can enhance tumor immunogenicity and improve responsiveness to immunotherapy. Mechanistically, we demonstrated that PP2A inhibition in tumor cells or tumor-associated macrophages activates innate immune sensing pathways, including cGAS/STING signaling, leading to induction of type I IFN responses and increased antigen presentation. Consistent with these findings, prior studies have shown that PP2A inactivation can promote tumor immunogenicity through additional mechanisms, including induction of microsatellite instability and neoantigen production (44) as well as perturbation of mRNA splicing that generates neoantigens (45). In addition, a growing body of work suggests that broad PP2A inhibition can induce cellular stress responses through paradoxical hyperactivation of oncogenic signaling pathways (16, 28). Together, these findings suggest that PP2A inhibition may promote antitumor immunity through both tumor-intrinsic and immune microenvironment–mediated mechanisms.

Supporting the clinical relevance of this concept, recent human studies demonstrate that tumors harboring endogenous PP2A-inactivating mutations, particularly *PPP2R1A* alterations in clear cell ovarian carcinoma, show markedly improved responses to immune checkpoint blockade, accompanied by increased IFN signaling and immune infiltration. *PPP2R1A* encodes the conserved PP2A scaffolding subunit required for holoenzyme assembly, and its mutation functionally disrupts PP2A signaling (46). Together, these findings suggest PP2A functions as a signaling rheostat whose modulation can produce context-dependent effects on tumor biology and antitumor immunity.

Consistent with this concept, our data indicate that PP2A inhibition exerts both tumor-intrinsic and immune-mediated antitumor effects. We observed direct tumor-suppressive activity following PP2A inhibition or genetic PP2Ac loss, consistent with induction of senescence and reduced tumor cell proliferation. At the same time, our syngeneic models reveal an additional immune-dependent component, in which senescence-associated immunogenic programs promote CD8^+^ T cell–mediated tumor control. Importantly, depletion of CD8^+^ T cells or loss of adaptive immunity significantly attenuated the survival benefit, indicating that immune-mediated tumor clearance contributes substantially to the therapeutic effect. These findings may also explain why prior xenograft studies, which lack an intact immune microenvironment, likely underestimate the full therapeutic potential of PP2A inhibition. These findings raise the possibility that combining PP2A inhibition with immune-based therapies, such as checkpoint blockade or adoptive T cell therapies, may further enhance therapeutic responses by promoting senescence-driven immune priming.

Despite these promising findings, translating PP2A inhibition into an effective therapy for brain tumors presents several challenges. Because PP2A is ubiquitously expressed, tumor-specific targeting is necessary to minimize systemic toxicity. In addition, clinical studies have shown that LB-100, a clinical-stage PP2A inhibitor, achieves limited penetration into brain tumors due to poor blood-brain barrier permeability. Emerging evidence from both prior studies and our current data suggest LB-100 exhibits modest potency and potential off-target effects, which may limit its therapeutic window. To address these limitations, we developed a localized nanotherapeutic strategy using LNPs encapsulating siRNA targeting PP2Ac. Direct intratumoral delivery of LNP-siPP2Ac recapitulated the effects of genetic PP2Ac deletion, achieving efficient local PP2A silencing, induction of senescence, and enhanced tumor immunogenicity. Several questions remain. The long-term fate of senescent tumor cells and their potential contribution to tumor recurrence require further investigation. In addition, the precise mechanisms linking PP2A inhibition to immune activation warrant deeper study, including the immune cell subsets responsible for tumor clearance and the signaling pathways connecting senescence to IFN activation. Although our study focuses on MB, these findings may extend to other p53-mutant malignancies, including high-grade glioma, pancreatic ductal adenocarcinoma, and triple-negative breast cancer, where induction of p53-independent senescence may provide therapeutic benefit.

In summary, this study identifies PP2A as a central regulator of immunogenic senescence in MB. Inhibition of PP2Ac induces a robust senescence program that enhances tumor immunogenicity and promotes CD8^+^ T cell–mediated tumor control. Leveraging this biology, we developed an LNP-based platform for targeted delivery of PP2Ac-directed siRNA that effectively reprograms the tumor microenvironment and prolongs survival in preclinical models. These findings provide a mechanistic framework for therapeutically harnessing immunogenic senescence and suggest targeting PP2A may represent a promising strategy for high-risk MB.

## Methods

### Sex as a biological variable

Both male and female mice were used in equal numbers across all animal experiments. For analyses of human MB datasets, both male and female patients were included. Sex was not evaluated as an independent biological variable in the primary statistical analyses; however, the findings reported here are expected to be relevant to both sexes.

### Cell lines

The murine MB cell line #2416 was provided by Martine Roussel (St. Jude Children’s Research Hospital, Memphis, Tennessee). Human MB cell lines D425 and CTD were obtained from Samuel Cheshier (University of Utah, Salt Lake City, Utah) and Jiyang Yu (St. Jude Children’s Research Hospital, Memphis, Tennessee), respectively. The D341 cell line was purchased from ATCC. All cell lines were routinely tested for *Mycoplasma* contamination.

D425 cells were cultured in Neurobasal-A/DMEM-F12 medium supplemented with B27, MEM nonessential amino acids, GlutaMAX, and growth factors, including EGF and FGF. We cultured #2416 cells in neurobasal medium supplemented with B27, N2, and growth factors. Cells were maintained in ultra-low attachment culture conditions to promote neurosphere formation and were dissociated with Accutase for passaging or experimental use. Detailed culture conditions and reagents are listed in Supplemental Table 2.

### CRISPR-mediated gene deletion

CRISPR/Cas9-mediated gene KO was performed in D425 and #2416 MB cells. For #2416, CTD and D341 cells, sgRNAs targeting *PPP2CA* were delivered using lentiviral lentiCRISPRv2 vectors. Viral particles were produced in HEK293A cells and used to transduce tumor cells, followed by puromycin selection. For D425 cells, sgRNAs targeting *PPP2CA* or *PPP2R5A* were cloned into PX459 vectors, followed by transient transfection and puromycin selection. Single-cell clones were expanded and validated for gene KO by immunoblotting. sgRNA sequences are listed in Supplemental Table 2.

### Inducible CRISPR/Cas9 model

Inducible Cas9 expression was established in D425 cells using lentiviral Edit-R inducible Cas9 particles (Dharmacon). Cas9 expression was induced using doxycycline and confirmed by immunoblotting. Cells were subsequently transduced with sgRNAs targeting *PPP2CA* or nontargeting control sgRNAs and selected with blasticidin and puromycin. KO efficiency and induction kinetics were confirmed by Western blot analysis.

### Animals

Male and female mice aged 8–10 weeks were used for experiments. C57BL/6J, NU/J, and Cd8atm1Mak/J mice were obtained from The Jackson Laboratory and maintained under specific pathogen–free conditions.

### In vivo experiments

#### Orthotopic brain tumor models.

Orthotopic MB tumors were established by stereotactic intracranial implantation of tumor cells into the brain of anesthetized mice. Briefly, 3 × 10^4^ tumor cells suspended in PBS were injected into the cerebellum using stereotactic guidance. Mice were monitored daily and euthanized upon reaching predefined neurological or weight-loss endpoints. For radiotherapy experiments, focal cranial irradiation was delivered using an X-ray irradiator while shielding noncranial tissues.

### In vitro experiments

#### Flow cytometry.

Cells were stained with fluorophore-conjugated antibodies in FACS buffer for 30 minutes at 4°C. Data were acquired using Cytek Aurora or BD Accuri cytometers and analyzed using FlowJo software. Antibody information is provided in Supplemental Table 2.

#### RT-qPCR.

Total RNA was isolated using a commercial RNA extraction kit. cDNA synthesis was performed using reverse transcription reagents, and gene expression was quantified using SYBR Green qPCR. Gene expression levels were normalized to GAPDH (human samples) or OAZ (mouse samples). Primer sequences are listed in Supplemental Table 2.

#### SA-β-Gal staining.

Cellular senescence was assessed by detection of SA-β-gal activity using a commercial senescence staining kit according to the manufacturer’s instructions. Cells were imaged by light microscopy, and senescent cells were quantified.

#### Immunofluorescence staining.

Tumor tissues were harvested, embedded in OCT, and cryosectioned. Sections were fixed, permeabilized, and incubated with primary antibodies against CD8 or senescence markers followed by fluorescent secondary antibodies. Nuclei were counterstained with DAPI. Images were acquired using confocal microscopy.

#### Quantification of immunofluorescence.

For D425 tumors, immunofluorescence staining for phosphorylated NF-κB (p-p65) and SA-β-gal was quantified using digital image analysis. Quantification was performed using QuPath Positive Cell Detection, in which 5 randomly selected 2,000 × 2,000 pixel regions of interest (ROIs) were analyzed per section to determine the proportion of positive cells. Values from multiple ROIs were averaged within each tumor, and statistical analyses were performed using tumor-level means. For #2416 tumors, image analysis was performed using Fiji. SA-β-gal signal intensity was quantified within tumor ROIs defined by RFP-positive tumor cells. CD8^+^ T cells were quantified as the number of positive cells per field of view. When multiple ROIs were analyzed per tumor, values were averaged within each tumor and statistical analyses were performed using tumor-level means.

#### Cytoplasmic dsDNA quantification.

D425 cells were irradiated at the indicated doses and harvested 24 hours later. For LNP-treated experiments, cells were pretreated with LNPs for 48 hours prior to irradiation. Cytoplasmic fractions were isolated using nuclear and cytoplasmic extraction reagents. Cytoplasmic dsDNA was quantified using a fluorescence-based dsDNA detection assay and normalized to live cell counts.

#### RNA-Seq.

Total RNA from WT and PP2Ac-deficient MB cells was extracted and subjected to RNA-Seq. Libraries were prepared and sequenced by Novogene. Differentially expressed genes were identified using thresholds of adjusted *P* < 0.05 and |log_2_ fold change| > 0.5. Pathway enrichment analyses were performed using Enrichr, and GSEA was conducted using MSigDB gene sets. Gene regulatory network and driver activity analyses were performed using the NetBID2 framework as previously described (24, 25), using the publicly available software package (https://github.com/jyyulab/NetBID; commit ID 8c1b11d21af96496bbfec1014aeb109c090339b2).

#### Immunoblotting.

Whole-cell lysates were prepared using RIPA buffer containing protease inhibitors. Protein concentrations were determined by bicinchoninic assay. Proteins were separated by SDS-PAGE and transferred to nitrocellulose membranes. Membranes were incubated with primary antibodies followed by HRP-conjugated secondary antibodies, and signals were detected using chemiluminescence. Antibody details are provided in Supplemental Table 2.

#### Cytokine array.

CM from irradiated D425 cells was collected and analyzed using a human cytokine array kit according to the manufacturer’s instructions.

#### PP2A regulatory subunit siRNA screen.

A library of validated siRNAs targeting known PP2A regulatory and scaffold subunits (Horizon Discovery) was transfected into D425 cells according to the manufacturer’s protocol. These siRNAs were prevalidated by the vendor for efficient target knockdown. siRNA sequence information is included in Supplemental Data 2. Forty-eight hours after transfection, *CDKN1A* (p21) expression was quantified by RT-qPCR as a readout of senescence induction.

#### Senescence score analysis.

Senescence scores were calculated for bulk transcriptomic samples using ssGSEA based on the previously published Fridman senescence gene signature (29). Scores were computed using the NetBID2 R package. To determine an unbiased cutoff for survival analysis in the GEO GSE85217 cohort, maximally selected rank statistics were applied to all patients with available survival information, yielding an optimal cutoff of 0.1350506. This cutoff was then used to stratify patients into senescence-high and senescence-low groups for Kaplan-Meier survival analysis. Survival analyses were performed using the survival and survminer R packages.

#### Human MB scRNA-Seq analysis.

Publicly available scRNA-Seq data from human MB tumors (GEO GSE155446) were analyzed using Seurat. Batch effects were corrected using Harmony integration. Senescence scores were calculated for individual tumor cells using curated senescence gene signatures, and cells were classified as high-senescence based on expression thresholds.

#### Gene regulatory network analysis.

A MB-specific gene regulatory network was constructed using SJARACNe based on bulk transcriptomic data from the GEO GSE85217 dataset. Network activity inference was performed using the scMINER package to estimate protein activity from single-cell expression data.

#### LNP synthesis and characterization.

LNPs encapsulating siPP2Ac were generated using an ethanol injection method with ionizable lipids. Particle-size distribution was measured by dynamic light scattering. Fluorescently labeled LNPs were used for uptake and intracellular trafficking studies in MB cells.

#### LNP uptake and intracellular trafficking.

MB cells were incubated with fluorescently labeled LNPs and analyzed by flow cytometry at defined time points to measure uptake kinetics. Confocal microscopy was used to visualize intracellular localization.

#### In vivo LNP treatment.

For therapeutic experiments, #2416 tumor cells were implanted intracranially in C57BL/6 mice. Guide cannulas were surgically implanted for intracranial delivery of LNP formulations. Mice received 250 μg siRNA/kg of control or PP2Ac-targeting LNPs at defined time points following tumor implantation. Survival was monitored according to institutional animal protocols.

Key experimental reagents, oligonucleotide sequences, and antibodies are listed in Supplemental Table 2.

### Statistics

For cell-based experiments, biological triplicates were generally performed unless otherwise specified. Animals were randomized into experimental groups after tumor implantation. Survival curves were generated using the Kaplan-Meier method and compared using the log-rank test. For comparisons between 2 groups, 2-tailed Student’s *t* tests were used. For comparisons across multiple groups, 1-way ANOVA was used. When multiple groups were compared with a shared reference group, 1-way ANOVA followed by Dunnett’s multiple-comparisons test was used. When all pairwise comparisons between groups were evaluated, 1-way ANOVA followed by Tukey’s multiple-comparisons test was used. Statistical analyses were performed using GraphPad Prism software. Data are presented as mean ± SEM unless otherwise stated. A *P* value of less than 0.05 was considered statistically significant.

### Study approval

All animal studies were approved by the Institutional Animal Care and Use Committees of UCSF and Northwestern University Feinberg School of Medicine.

### Data availability

Publicly available datasets analyzed in this study include GEO GSE85217 and GSE155446. Sequencing data generated in this study are available in the GEO under accession GSE302307. All other data generated in this study are provided in the Supporting Data Values file or are available from the corresponding author upon reasonable request. Supporting data values underlying all figures are provided in an XLS file.

## Author contributions

WSH and ROL conceived the project and designed the experiments. WSH, IM, RS, CG, DT, and OD performed the majority of experiments, with assistance from all authors. JL and JY conducted all bioinformatic analyses. JH, JS, HL, and PZ performed LNP synthesis and characterization, and contributed to in vitro and in vivo studies.

## Conflict of interest

The Regents of the University of California have filed a patent application related to PP2A-targeting lipid nanoparticle therapeutics (LNP-Encapsulated Oligonucleotides for Targeting PP2A in Cancer; U.S. Patent Application No. 63/875,748, filed September 4, 2025), on which WSH, PZ, and ROL are listed as inventors. WSH and ROL are also inventors on an issued patent related to PP2A modulation in immunotherapy (Oxabicycloheptanes for modulation of immune response, patent US20200069680A1).

## Funding support

This work is the result of NIH funding, in whole or in part, and is subject to the NIH Public Access Policy. Through acceptance of this federal funding, the NIH has been given a right to make the work publicly available in PubMed Central.

- NIH National Institute of Neurological Disorders and Stroke (grants R01NS126501 to ROL and R01NS131545 to WSH).
- NIH National Cancer Institute (grant 5R37CA266487 to PZ).
- Meghan Rose Bradley Foundation (to WSH).
- Alex’s Lemonade Stand Foundation for Childhood Cancer (to WSH).
- Matthew Larson Foundation for Pediatric Brain Tumors (to WSH).

## Supplementary Material

Supplemental data

Supplemental data sets 1-2

Unedited blot and gel images

Supporting data values

## Version 1 05/05/2026

In-Press Preview

## Version 2 07/01/2026

Electronic publication
