# Supplementary material for: Protein phosphatase 2A regulates senescence and immunogenicity in medulloblastoma models
Source: J Clin Invest. 2026 Apr 23;136(13):e196753. doi: 10.1172/JCI196753 (PMC13318113; doi:10.1172/JCI196753)
Supplement: Supplemental data [file jci-136-196753-s033.pdf]

Supplementary Methods

Lentiviral particles were produced by transient transfection of HEK293A cells with transfer plasmids together with the packaging plasmids psPAX2 and pMD2.G using Lipofectamine 3000 (Thermo Fisher Scientific). Viral supernatants were collected 48 hours after transfection, filtered through a 0.45 μm filter, and used to transduce medulloblastoma cells in the presence of polybrene to enhance infection efficiency. Transduction was further facilitated by centrifugation ("spinoculation"). Following transduction, cells were selected with puromycin to generate stable knockout or control cell populations.

D425 and D341 medulloblastoma cells were cultured in a 1:1 mixture of Neurobasal-A and DMEM/F12 medium supplemented with B27, MEM non-essential amino acids, GlutaMAX, sodium pyruvate, sodium bicarbonate, penicillin–streptomycin, HEPES, epidermal growth factor (EGF), fibroblast growth factor (FGF), heparin, and leukemia inhibitory factor (LIF). Murine #2416 and CTD medulloblastoma cells were cultured in Neurobasal medium supplemented with B27, N2, GlutaMAX, EGF, and FGF. Cells were maintained in ultra-low attachment flasks to promote neurosphere growth and were dissociated with Accutase for passaging or experimental use. Detailed reagent sources and media components are listed in Table S2.

For flow cytometry experiments, cells were harvested and washed with FACS buffer consisting of phosphate-buffered saline supplemented with fetal bovine serum and EDTA. Cells were incubated with fluorophore-conjugated antibodies for 30 minutes at 4°C in the dark. Following staining, cells were washed and resuspended in FACS buffer for analysis. Data were acquired using Cytek Aurora or BD Accuri cytometers and analyzed using FlowJo software. Antibodies and reagents used for flow cytometry experiments are listed in Table S2.

For immunofluorescence experiments, tumor tissues were embedded in OCT compound and cryosectioned into 10 μm sections. Sections were fixed with paraformaldehyde, washed with PBS, and

permeabilized using detergent-containing buffer. Samples were blocked with serum-containing blocking buffer and incubated overnight at 4°C with primary antibodies. Following washing steps, samples were incubated with fluorophore-conjugated secondary antibodies for 1 hour at room temperature. Nuclei were counterstained with DAPI and slides were mounted with antifade mounting medium. Images were acquired using confocal microscopy. Antibodies used for immunofluorescence staining are listed in Table S2.

Conditioned media from treated D425 cells were collected and clarified by centrifugation prior to analysis. Cytokine secretion was measured using a human cytokine array kit according to the manufacturer's instructions. Membranes were incubated with conditioned media followed by chemiluminescent detection reagents. Signals were visualized using a digital imaging system and quantified relative to control conditions.

Lipid nanoparticles encapsulating PP2Ac-targeting siRNA were synthesized using an ethanol injection method. Ionizable lipid, cholesterol, phospholipid, and PEG-lipid components were dissolved in ethanol and rapidly mixed with siRNA diluted in acidic citrate buffer. Following nanoparticle formation, samples were neutralized and buffer exchanged into DPBS. Particle size distribution was measured using dynamic light scattering. For uptake and trafficking experiments, fluorescent lipids were incorporated into the nanoparticle formulation.

For nanoparticle uptake assays, medulloblastoma cells were incubated with fluorescently labeled lipid nanoparticles and analyzed by flow cytometry at defined time points to measure cellular uptake. For intracellular trafficking experiments, cells were cultured on glass-bottom dishes and incubated with labeled nanoparticles followed by membrane and nuclear staining. Cells were imaged using confocal microscopy to visualize nanoparticle localization.

Bulk RNA sequencing data were analyzed using standard RNA-seq analysis pipelines. Differential gene expression between WT and PP2Ac-deficient medulloblastoma cells was determined using normalized expression values. Genes with an adjusted P value less than 0.05 and absolute log2 fold change greater than 0.5 were considered differentially expressed. Pathway enrichment analysis was performed using Enrichr, and gene set enrichment analysis (GSEA) was conducted using the GSEA software package (version 4.0.3) with gene sets from the Molecular Signatures Database (MSigDB version 7.1). Genes were ranked according to log2 fold change for preranked GSEA analysis.

Gene regulatory network construction and driver activity inference were performed using the NetBID2 framework as previously described. A medulloblastoma-specific regulatory network was constructed using transcriptomic data from the publicly available medulloblastoma cohort GSE85217. Driver activity scores were calculated using functions implemented in the NetBID2 software package (https://github.com/jyyulab/NetBID).

Senescence scores were calculated using previously published senescence gene signatures including the Fridman_UP, Fridman_DOWN, and Casella_UP gene sets. For bulk RNA-seq data, senescence scores were calculated using single-sample gene set enrichment analysis by subtracting the enrichment score of the downregulated signature from that of the upregulated signature. To evaluate the association between senescence score and overall survival in the GSE85217 cohort, senescence score was first analyzed as a continuous variable using a Cox proportional hazards model. To determine an unbiased cutoff for categorical survival analysis, maximally selected rank statistics were applied to all patients with available survival information. The resulting cutoff was used to stratify patients into senescence-high and senescence-low groups for Kaplan–Meier survival analysis and Cox proportional hazards modeling. Survival analyses were performed using the survival and survminer R packages.

Single-cell RNA sequencing data from human medulloblastoma samples were obtained from the Gene Expression Omnibus dataset GSE155446. Data were processed and analyzed using the Seurat R package. Batch effects between samples were corrected using the Harmony integration algorithm.

Senescence scores were calculated for individual cells using curated senescence gene signatures normalized to total transcript counts per cell. Cells were classified as high-senescence malignant cells based on expression thresholds for senescence-associated signatures. Dimensionality reduction and clustering analyses were performed using UMAP according to standard Seurat workflows.

Supplementary Figures

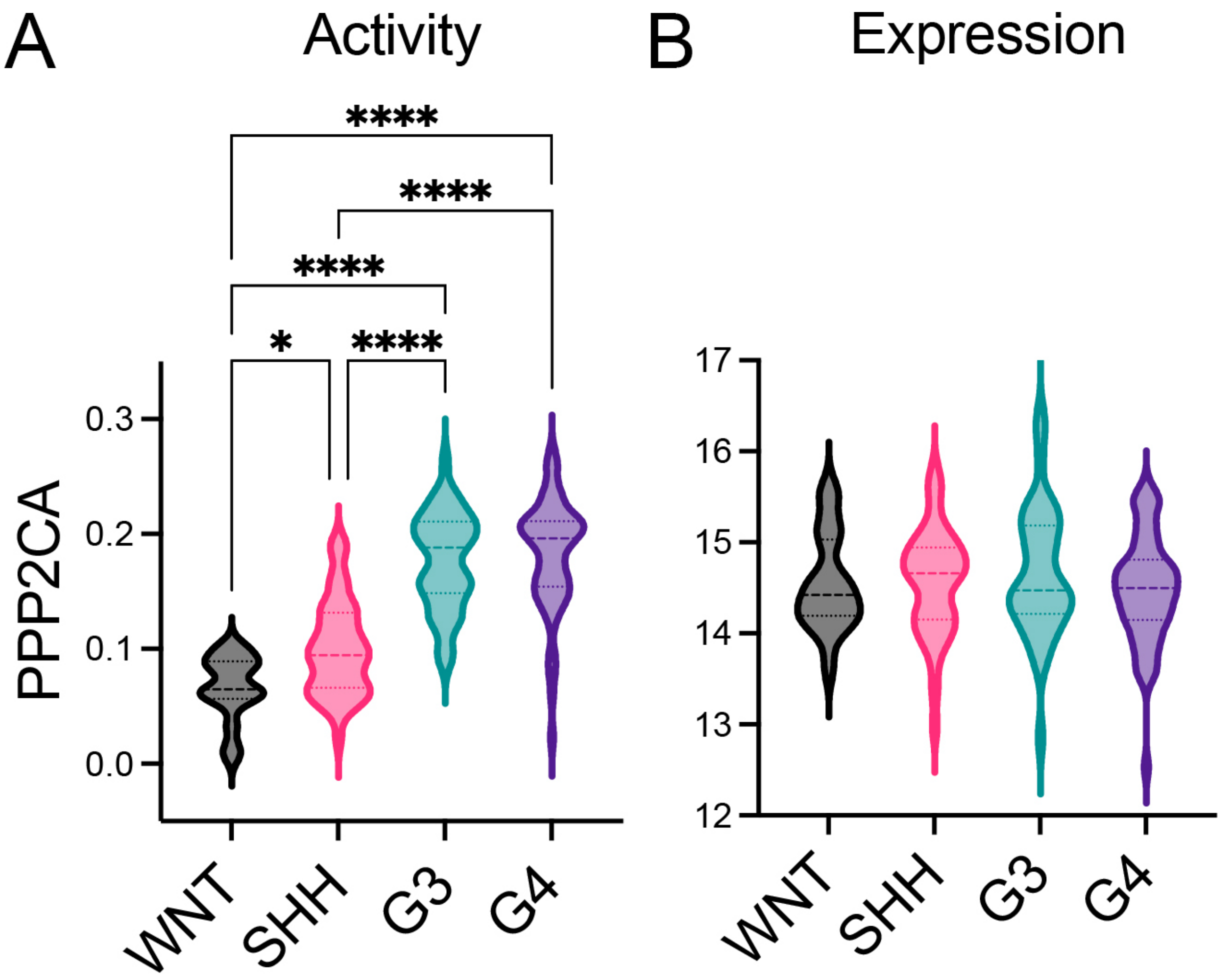

Figure S1. PP2Ac activity is enriched in Group 3 and Group 4 medulloblastoma. (A) NetBID2-inferred PP2Ac activity across medulloblastoma molecular subgroups using a publicly available dataset of 169 human MB samples[1]. (B) RNA expression of PPP2CA across MB subgroups from the same dataset. Statistical significance was determined using one-way ANOVA (*P < 0.05, ****P < 0.001).

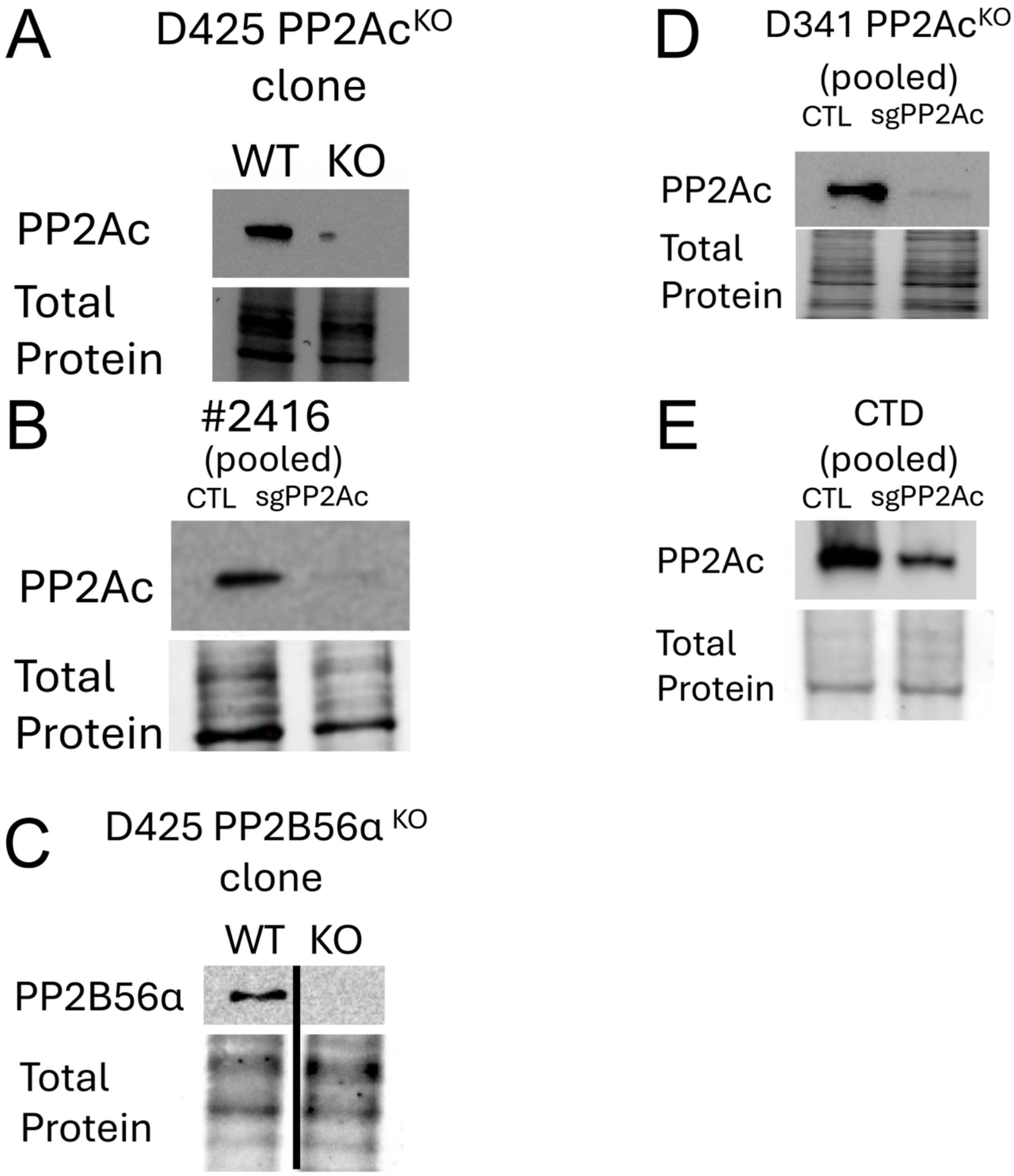

Figure S2. Western blot confirmation of genetically modified cell lines. (A) D425 PP2Ac-KO clone. (B) #2416 pooled PP2Ac-KO. (C) D425 PP2B56a-KO clone. (D) D341 pooled PP2Ac-KO. (E) CTD pooled PP2Ac-KO. Lanes in (C) separated by black vertical line were run on the same gel but were noncontiguous.

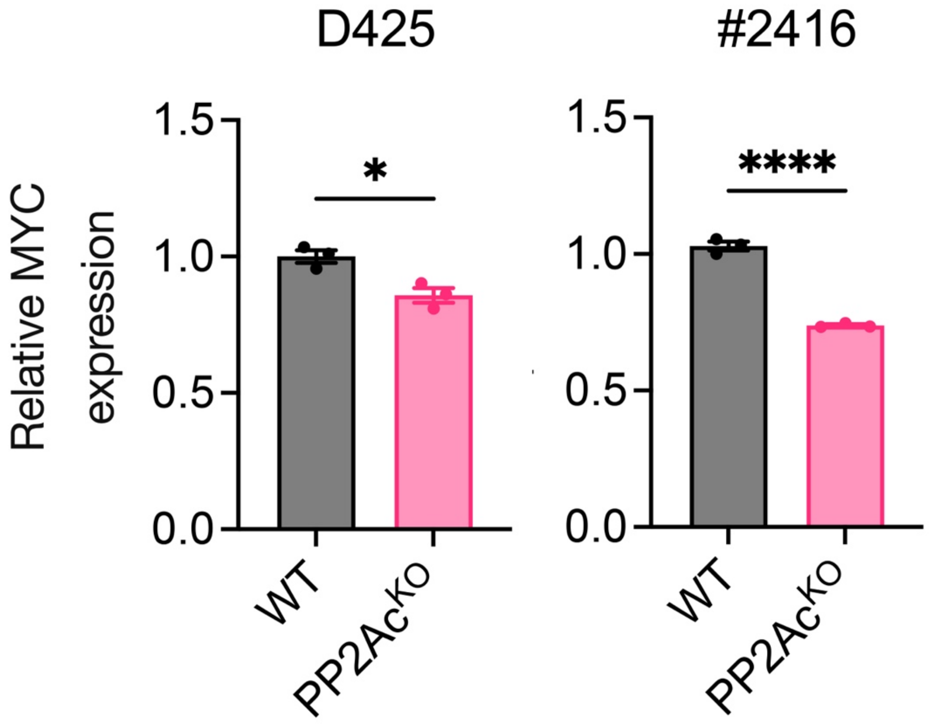

Figure S3. PP2Ac loss reduces MYC expression across multiple Group 3 medulloblastoma models. RNA-seq analysis of MYC expression in D425 and #2416 medulloblastoma cells following PP2Ac knockout. Expression values were derived from log2(FPKM) RNA-seq data and normalized to the mean expression of the corresponding WT control. Each dot represents an individual biological replicate (n = 3 per group). Data represent mean ± SEM. Statistical significance was determined using a two-tailed unpaired t test. P < 0.05 was considered statistically significant (*P < 0.05, ***P < 0.0001).

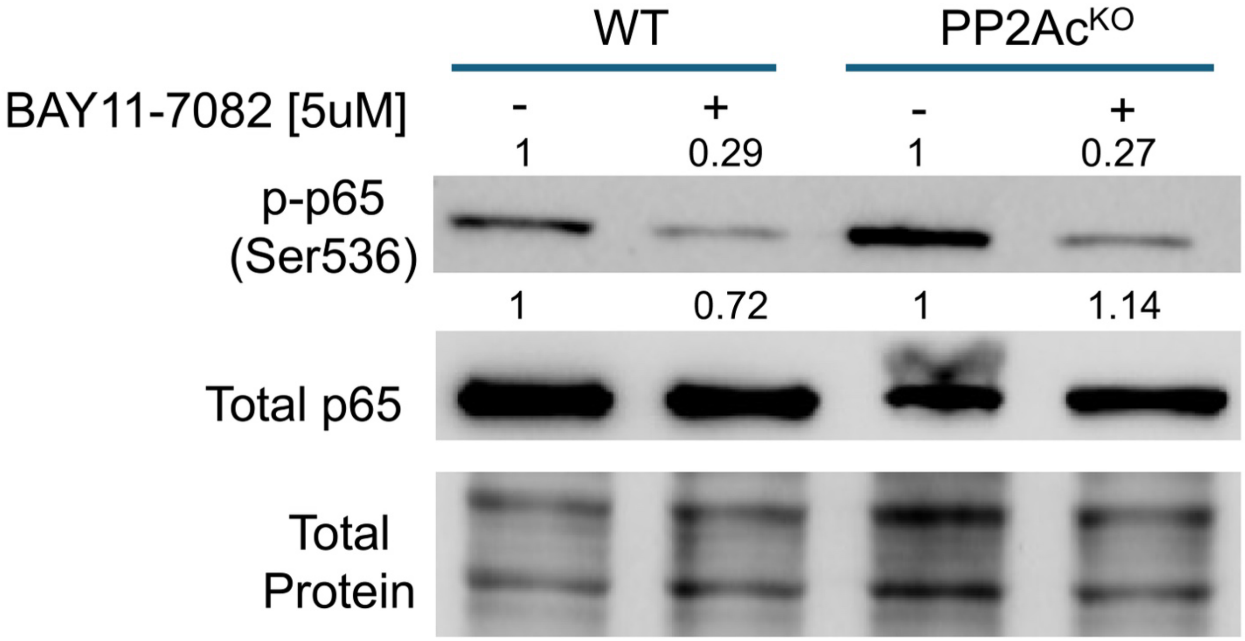

Figure S4. Bay11-7082 effectively inhibits NF-κB activation in medulloblastoma cells. Immunoblot analysis of phosphorylated p65 (Ser536) and total p65 in WT and PP2AcKO D425 cells 1 hour after treatment with the NF-κB inhibitor Bay11-7082 (5 μM). Bay11-7082 treatment markedly reduced p-p65 (Ser536) levels in both WT and PP2Ac-deficient cells, whereas total p65 levels remained largely unchanged, confirming effective inhibition of NF-κB signaling. Total protein staining was used as a loading control. Numbers above the bands indicate densitometric quantification normalized to the untreated condition within each genotype.

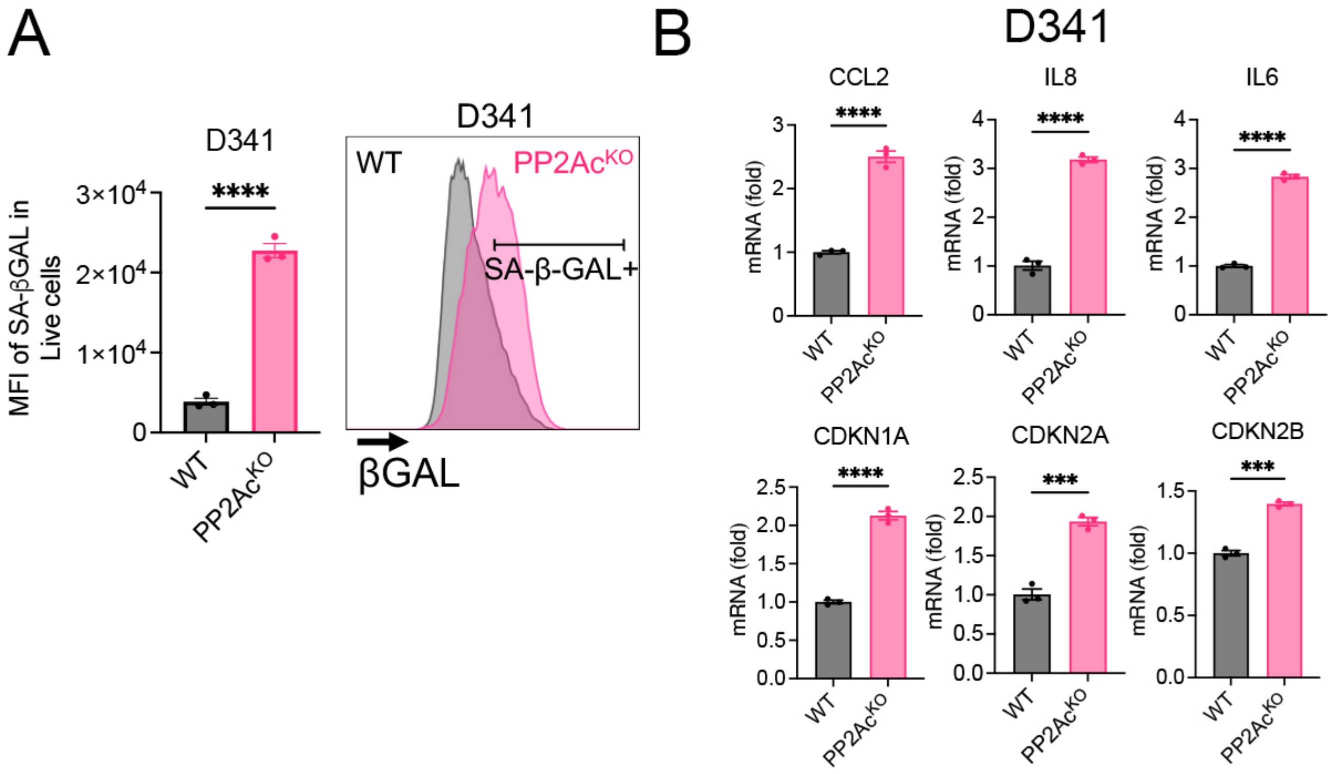

Figure S5. Loss of PP2Ac induces cellular senescence in D341 medulloblastoma cells. (A) Flow cytometric quantification of SA-β-gal activity in WT and PP2Ac-KO D341 cells. (B) RT–qPCR analysis of senescence-associated genes, including CDKN1A, IL8, IL6, CDKN2A, and CDKN2B, in WT and PP2Ac-KO D341 cells. Data are shown as mean ± SEM. Statistical significance was determined using unpaired 2-tailed t tests. $P < 0.05$ was considered statistically significant (***$P < 0.001$, ****$P < 0.0001$).

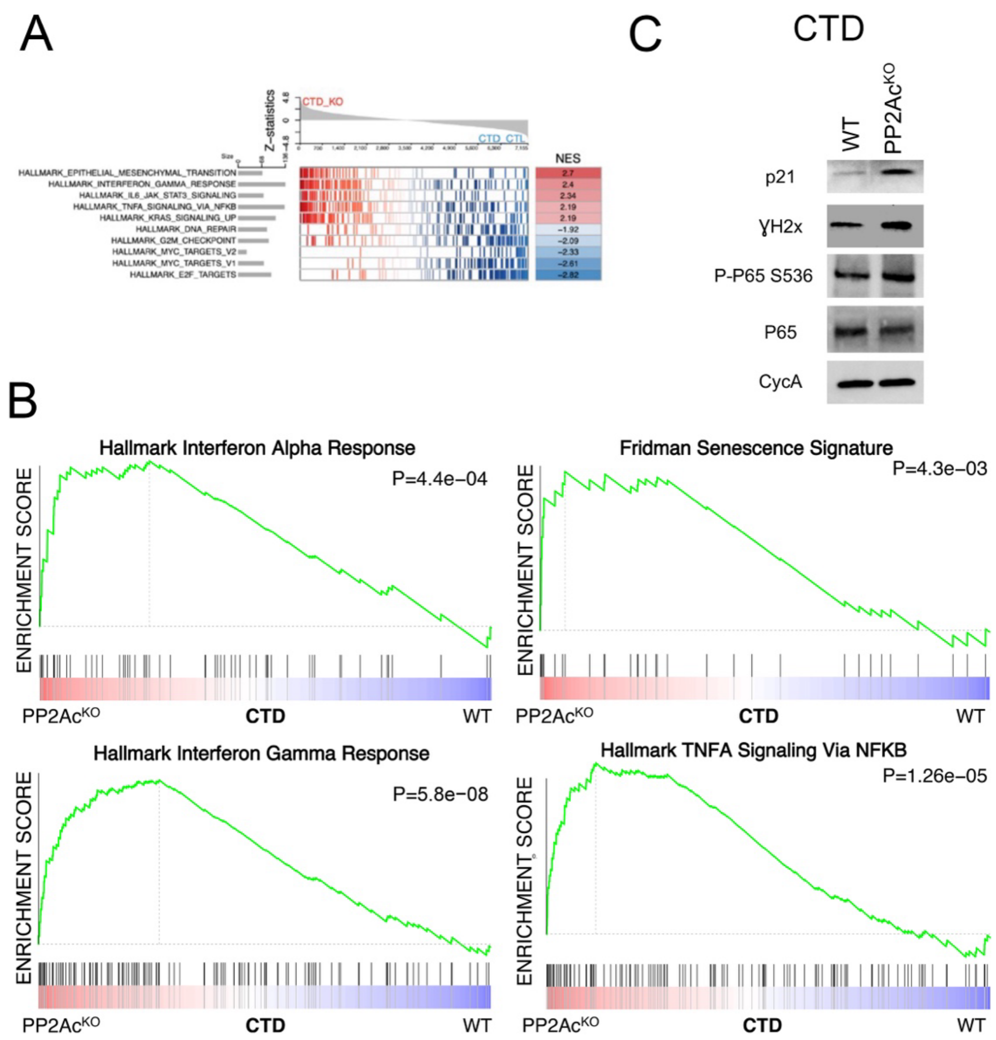

Figure S6. PP2Ac loss activates senescence-associated transcriptional programs in CTD medulloblastoma cells. (A) Hallmark pathway enrichment analysis using NetBID2-inferred protein activity profiles comparing WT and PP2Ac-KO CTD cells. Pathways are ranked by normalized enrichment score (NES), highlighting enrichment of inflammatory and stress-response programs. (B) Gene set enrichment analysis (GSEA) of NetBID2-inferred activity signatures demonstrating enrichment of Hallmark interferon-α response, interferon-γ response, TNFα signaling via NF-κB, and the Fridman senescence signature in PP2Ac-KO CTD cells relative to WT cells. (C) Immunoblot validation in CTD cells showing increased expression of p21, γH2AX, and phosphorylated p65 (p-p65, Ser536) in PP2Ac-KO cells compared with WT cells. Total p65 and cyclin A (CycA) are shown as controls.

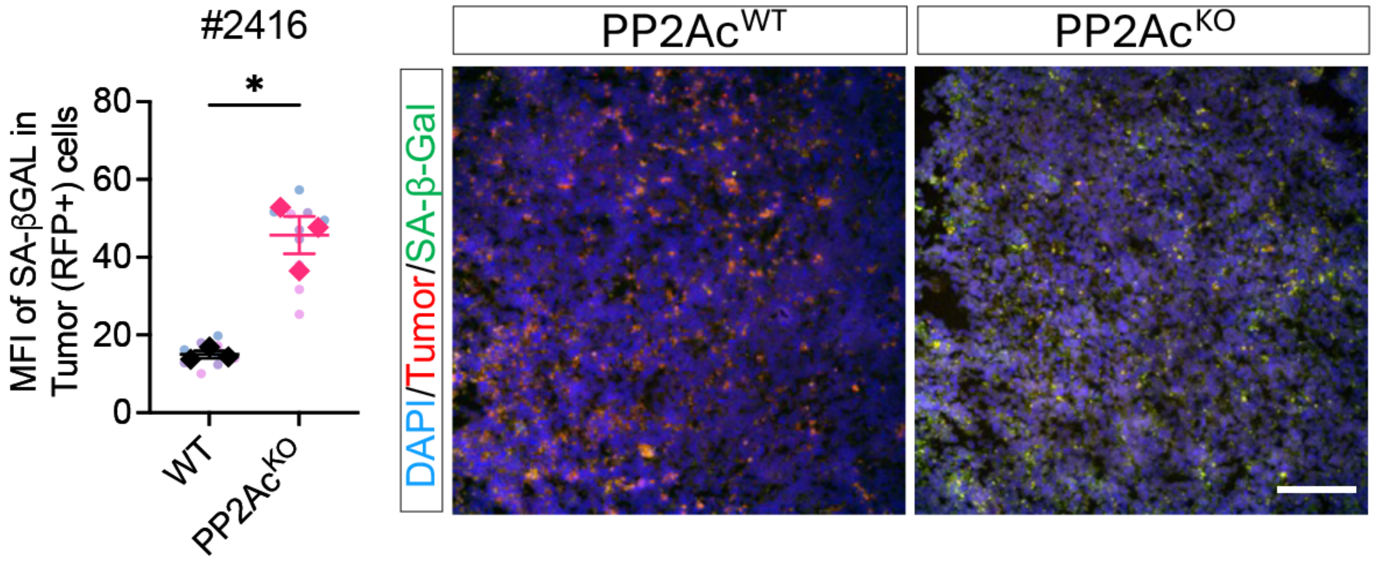

Figure S7. Loss of PP2Ac increases tumor senescence *in vivo*. Representative immunofluorescence images and quantification of SA-β-gal in orthotopic #2416 tumors collected at endpoint. SA-β-gal (green), tumor cells (RFP, red), and nuclei (DAPI, blue). SA-β-gal signal was quantified as mean fluorescence intensity (MFI) within RFP-positive tumor cells. Small colored dots represent individual ROIs nested within each biological replicate, and the larger dot represents the mean value for each tumor. Statistical analysis was performed using tumor-level means. Scale bar, 20 µm. Data are shown as mean ± SEM. Statistical significance was determined using unpaired 2-tailed t tests. $P < 0.05$ was considered statistically significant (*$P < 0.05$).

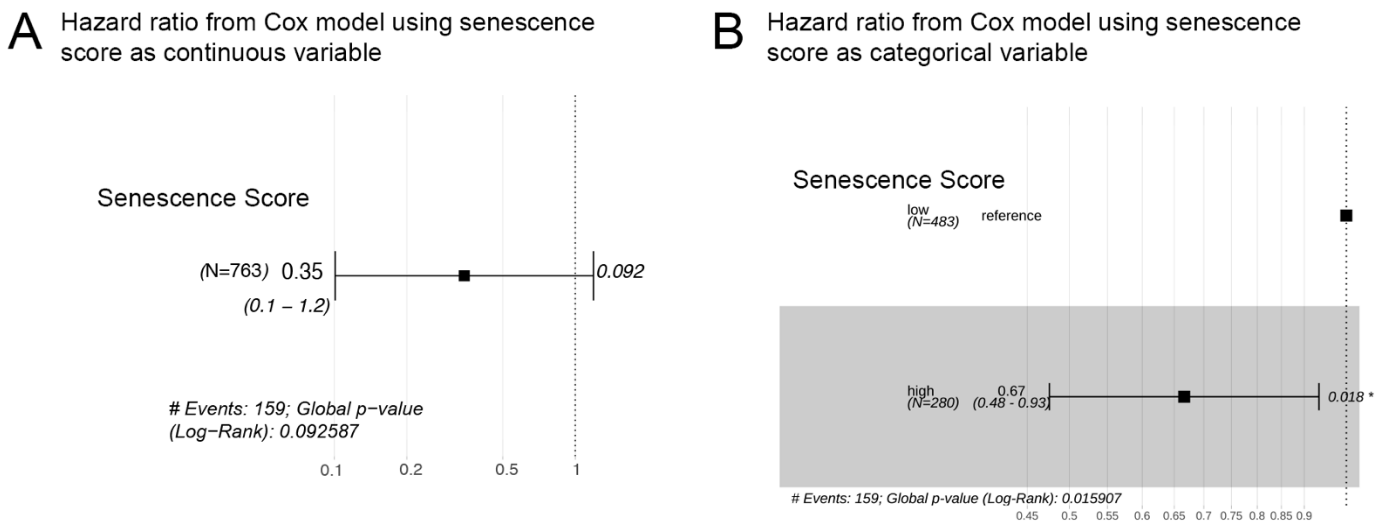

Figure S8. Cox proportional hazards analyses of senescence score and overall survival in medulloblastoma. (A) Forest plot showing the hazard ratio from a Cox proportional hazards model using senescence score as a continuous variable in the GSE85217 cohort. (B) Forest plot showing the hazard ratio from a Cox proportional hazards model using senescence score as a categorical variable, with patients stratified into senescence-high and senescence-low groups using the cutoff identified by maximally selected rank statistics. The senescence-low group was used as the reference. Hazard ratios are shown with 95% confidence intervals. N indicates the number of patients included in each analysis.

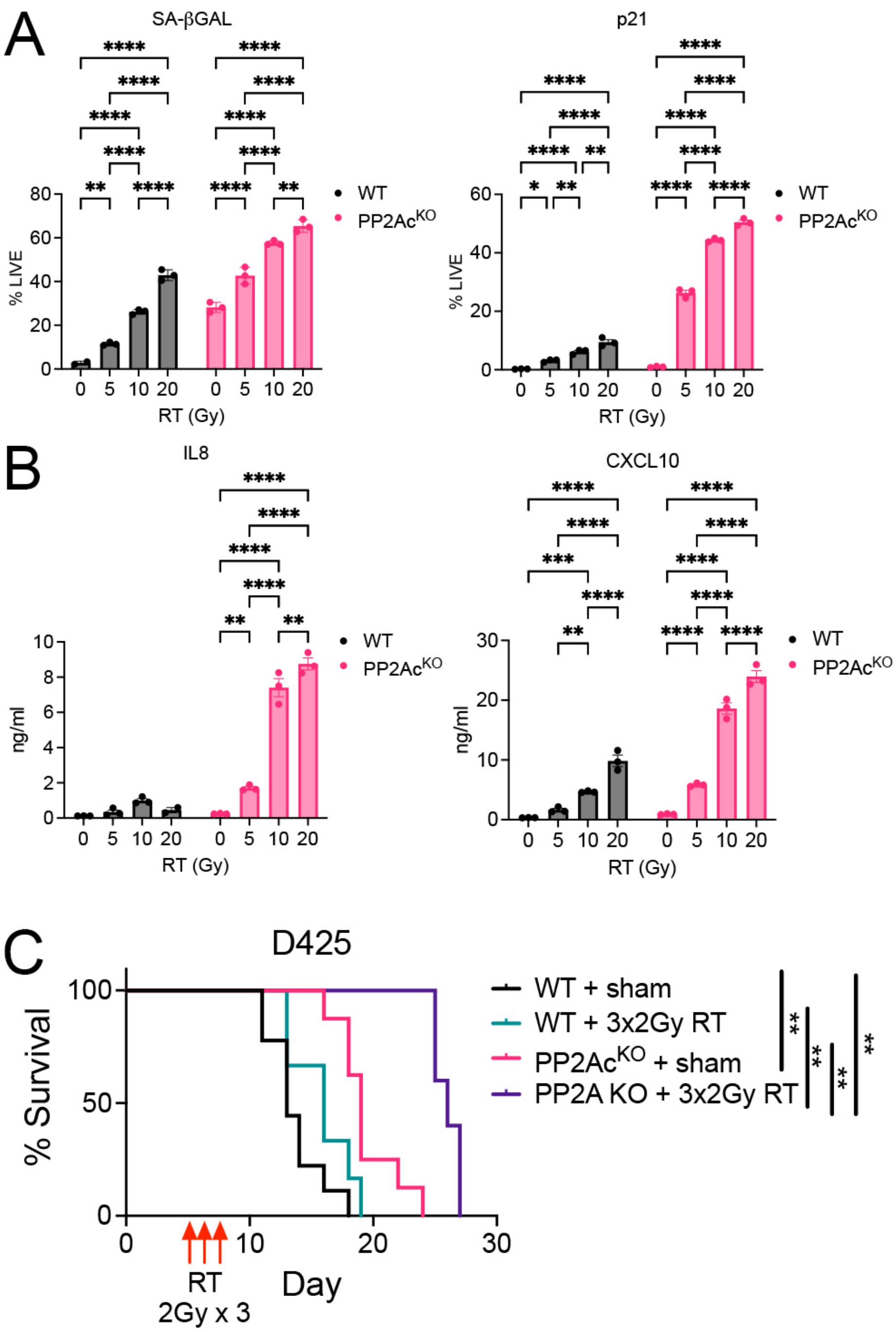

Figure S9. PP2Ac deficiency enhances radiation-induced senescence and inflammatory cytokine production. D425 WT and PP2Ac-KO cells were treated with ionizing radiation (RT). (A) Forty-eight hours after irradiation, SA-β-gal activity and p21 expression were quantified by flow cytometry. (B) Cytokine levels (CXCL10 and IL-8) in culture supernatants were measured by bead-based immunoassay 48 hours after irradiation. (C) Kaplan–Meier survival analysis of nude mice implanted intracranially with WT or PP2Ac-KO D425 cells followed by focal brain irradiation (20 Gy × 3). Data are shown as mean ± SEM. Statistical significance was determined using 1-way ANOVA followed by Tukey's multiple-comparisons test, or the log-rank test, as appropriate for each panel. $P < 0.05$ was considered statistically significant (*$P < 0.05$, **$P < 0.01$, ***$P < 0.001$, ****$P < 0.0001$).

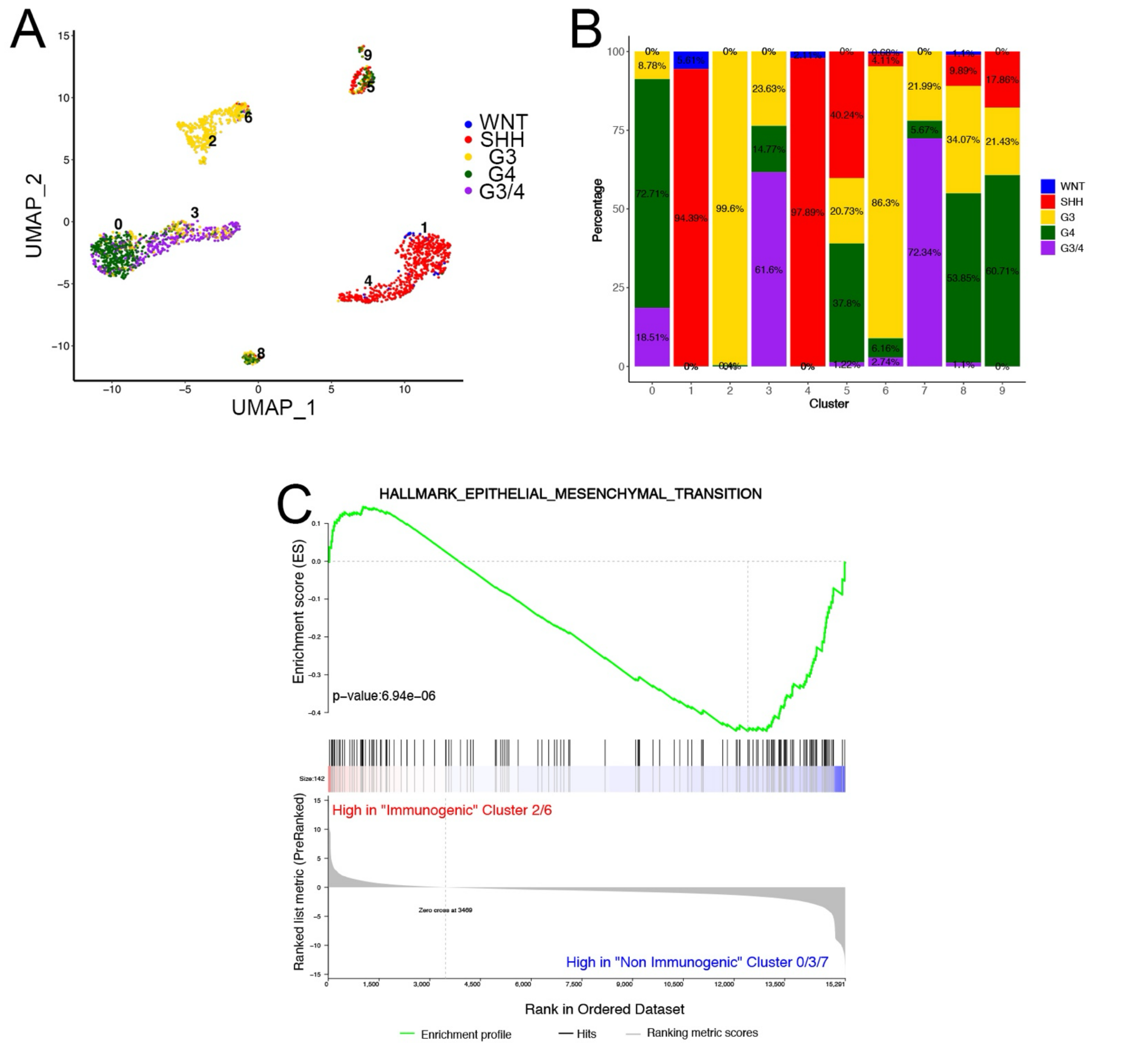

Figure S10. (A) UMAP visualization of 2,231 senescent MB single cells, color-coded by molecular subgroup (WNT, SHH, Group 3 [G3], Group 4 [G4], and intermediate G3/G4). (B) Distribution of MB subgroups across identified transcriptional clusters. Bar height indicates the proportion of each subgroup within a given cluster. (C) Gene Set Enrichment Analysis (GSEA) plot demonstrating significant enrichment of the epithelial–mesenchymal transition (EMT) hallmark signature in "immunogenic" clusters (Clusters 2 and 6) compared to "non-immunogenic" clusters (Clusters 0, 3, and 7).

**Fridman_UP**		**Fridman_DOWN**	**Casella_UP**	
CYRAB	*IRF5*	*ALDH1A1*	*TMEM159*	*TM4SF1-AS1*
IGFBP7	*TSPYL5*	*BMI1*	*CHPF2*	*PTCHD4*
SERPINE1	*MAP2K3*	*CCN4*	*SLC9A7*	*H2AJ*
CDKN1A	*RBL2*	*CCNB1*	*PLOD1*	*PURPL*
MMP1	*HPS5*	*CDC25B*	*FAM234B*	
ISG15	*IGFBP3*	*COL3A1*	*DHRS7*	
IGFBP6	*RGL2*	*E2F4*	*SRPX*	
MDM2	*RAB31*	*EGR1*	*SRPX2*	
IGFBP5	*TES*	*ID1*	*TNFSF13B*	
IGFBP1	*SOD1*	*LAMA1*	*PDLIM1*	
OPTN	*TP53*	*LDB2*	*ELMOD1*	
FN1	*HBS1L*	*MARCKS*	*CCND3*	
CCND1	*ING1*		*TMEM30A*	
PEA15	*TGFB1I1*		*STAT1*	
GSN	*IGFBP2*		*RND3*	
SMPD1	*CCN2*		*TMEM59*	
NRG1	*F3*		*SARAF*	
RRAS	*IRF7*		*SLCO2B1*	
IGFBP4	*HTATIP2*		*ARRDC4*	
GUK1	*CDKN2D*		*PAM*	
MAP1LC3B	*EIF2S2*		*WDR78*	
RAB5B	*SMURF2*		*WDR63*	
SPARC	*IGSF3*		*NCSTN*	
TNFAIP2	*CYP1B1*		*SLC16A14*	
RAB13	*TFAP2A*		*GPR155*	
THBS1	*ALDH1A3*		*CLDN1*	
CD44	*RHOB*		*JCAD*	
IFI16	*CDKN1C*		*BLCAP*	
STAT1	*CDKN2B*		*FILIP1L*	
TNFAIP3	*CDKN2A*		*TAP1*	
CREG1			*TNFRSF10C*	
CLTB			*SAMD9L*	
S100A11			*SMCO3*	
CITED2			*POFUT2*	
RAC1			*KIAA1671*	
HSPA2			*LRP10*	
VIM			*DIO2*	
RABGGTA			*MAP4K3-DT*	
AOPEP			*LINC02154*	

Table S1. Published senescence signature

Western Blot Antibodies			
Target Protein	Company	Human (cat#)	Mouse (cat#))
p-p65 (Ser536)	CST	3033	
Total p65	CST	8242	
Vinculin	CST	4650	
Actin	CST	8457	
c-myc	CST	5605	
P21	CST	2947	64016
Gh2ax	CST	9718	

Immunofluorescence Antibodies		
Target Protein	Company	Cat#
CD8 antibody	Abcam	ab217344 – to mouse tissue only
p-p65 (Ser536)	CST	3033
Goat Anti-Rabbit IgG H&L (Alexa Fluor® 488)	Abcam	ab150077 – ***secondary***
ImaGene Green™ C12FDG lacZ Gene Expression Kit	ThermoFisher	I2904
Senescence β-Galactosidase Staining Kit	CST	9860

Flow Cytometry Antibodies		
Target	Company	Cat#
Alexa Flour 488 Goat anti-rabbit IgG (H+L)	Invitrogen	A11008 – ***secondary***
P21	CST	2947 – human only
Alexa Fluor 594 goat anti-rabbit IgG (H+L)	Invitrogen	A11012 – secondary for P21
CellEvent™ Senescence Green Detection Kit	Thermo	C10851
APC anti-mouse H-2Kb/H-2Db, clone 28-8-6, Isotype Mouse C3H, IgG2a	BioLegend	114614
Brilliant Violet 510 anti-human HLA-A,B,C, clone W6/32, Isotype Mouse IgG2a	BioLegend	311436

Other (Inhibitors, Antibodies, Drugs, Cell Culture Medium Factors, etc.):			
Name	Target/Purpose	Company	Cat#
BAY 11-7082	NF-κB, inhibitor	Sigma	196870-10MG
LB-100	PP2A-c inhibitor	Selleck Chemicals	S7537
Doxycycline	Tet for cas9-inducible model	Fisher Scientific	ICN19895510
Puromycin	Selection for sgRNA	InvivoGen	ant-pr-1
Blasticidin S	Selection for induc-cas9 plasmid	InvivoGen	ant-bl-1
Anti-mouse CD8a (2.43)		BioXcell	BE0061
Ctrl IgG (Rat IgG2b control for anti-CD8a)		BioXcell	BE0090
SpectraMax® Quant™ AccuClear™ Nano dsDNA Assay Kit	Cytoplasmic dsDNA quantification	Molecular Devices	R8357
Cell Culture			

Neurobasal Media – A	D425	ThermoFisher	10888022
DMEM/F12		ThermoFisher	11320033
MEM NEAA		ThermoFisher	11140050
GlutaMAX-I		ThermoFisher	35050061
Sodium Pyruvate		ThermoFisher	11360070
Sod. Bicarbonate		ThermoFisher	25080094
HEPES		ThermoFisher	15630080
Pen/Strep		ThermoFisher	15140122
B27-A		Thermo	12587010
hEGF		Shenandoah	100-26
Basic FGF		Shenandoah	100-146
Heparin		Stemcell	07980
LIF		Peprotech	AF-300-05-100ug
Ultra low attachment T75 flask	#2416	Fisher	3814
Ultra low attachment T25 flask		Fisher	3815
Neurobasal medium		Invitrogen	21103-049
B27 (50x)		Invitrogen	175-04-044
N2 (100x)		Invitrogen	175-02-048
Human bFGF		PeproTech	AF-100-18B
Human EGF		PeproTech	AF-100-15
Accutase		Invitrogen	A11105-01

**qPCR Primer Sequences**		
Gene	Human – F	Human - R
GAPDH	GAAGGTGAAGGTCGGAGTC	GAAGATGGTGATGGGATTTC
CDKN1A	TCACTGTCTTGTACCCTTGTG	GGCGTTTGGAGTGGTAGAAA
CDKN2A	GAGCAGCATGGAGCCTTC	CGTAACTATTCGGTGCGTTG
CDKN2B	GGCAGTCGATGCGTTCACT	AGGGCCTAAGTTGTGGGTTCA
IGFBP6	GATGTGAACCGCAGAGACC	CCAGATGTCTACGGCATGG
TNF	GAGGCCAAGCCCTGGTATG	CGGGCCGATTGATCTCAGC
CXCL10	GTGGCATTCAAGGAGTACCTC	TGATGGCCTTCGATTCTGGATT
CCL2	AGTCTCTGCCGCCCTTCT	GTGACTGGGGCATTGATTG
IL8	TTTTGCCAAGGAGTGCTAAAGA	AACCCTCTGCACCCAGTTTTC

Gene	Mouse – F	Mouse – R
OAZ	TTATTGCTGTTTAAGATGGTCAG	GAACGAGATCACTTTATTGGATT
CDKN1A	CCTGGTGATGTCCGACCTG	CCATGAGCGCATCGCAATC
CDKN2A	GAACTCTTTCGGTCGTACCC	AGTTCGAATCTGCACCGTAGT
CDKN2B	CCCTGCCACCCTTACCAGA	GCAGATACCTCGCAATGTCAC
IGFBP6	TGCTAATGCTGTTGTTCGCTG	CACGGTTGTCCCTCTCTCCT
TNF	CAGGCGGTGCCTATGTCTC	CGATCACCCCGAAGTTCAGTAG
CXCL10	CCAAGTGCTGCCGTCATTTTC	GGCTCGCAGGGATGATTTCAA
CCL2	TTAAAAACCTGGATCGGAACCAA	GCATTAGCTTCAGATTTACGGGT
IL8	CTGTTAAATCTGGCAACCCTAGTCT	CAAGGCACAGTGGAACAAGGA

**sgRNA sequences**		
Gene	Species	Forward Seq, 5' to 3'
PP2Ac	Human	AACGCATCACCATTCTTCGA
PP2Ac	Mouse	ACATCGAACCTCTTGAACGT
PPP2R5A	Human	CAATGCTAGGCTGGAAATCA

siRNA for LNP		
Gene	Company	siRNA ID/catalog ID
PP2Ac	Sigma	SASI_Mm01_00038232
Non-targeting	Thermo	AM4611

Table S2. Key experimental reagents.
