# Supplementary material for: Protein phosphatase 2A regulates senescence and immunogenicity in medulloblastoma models
Source: J Clin Invest. 2026 Apr 23;136(13):e196753. doi: 10.1172/JCI196753 (PMC13318113; doi:10.1172/JCI196753)

Full unedited gel for Figure 2F

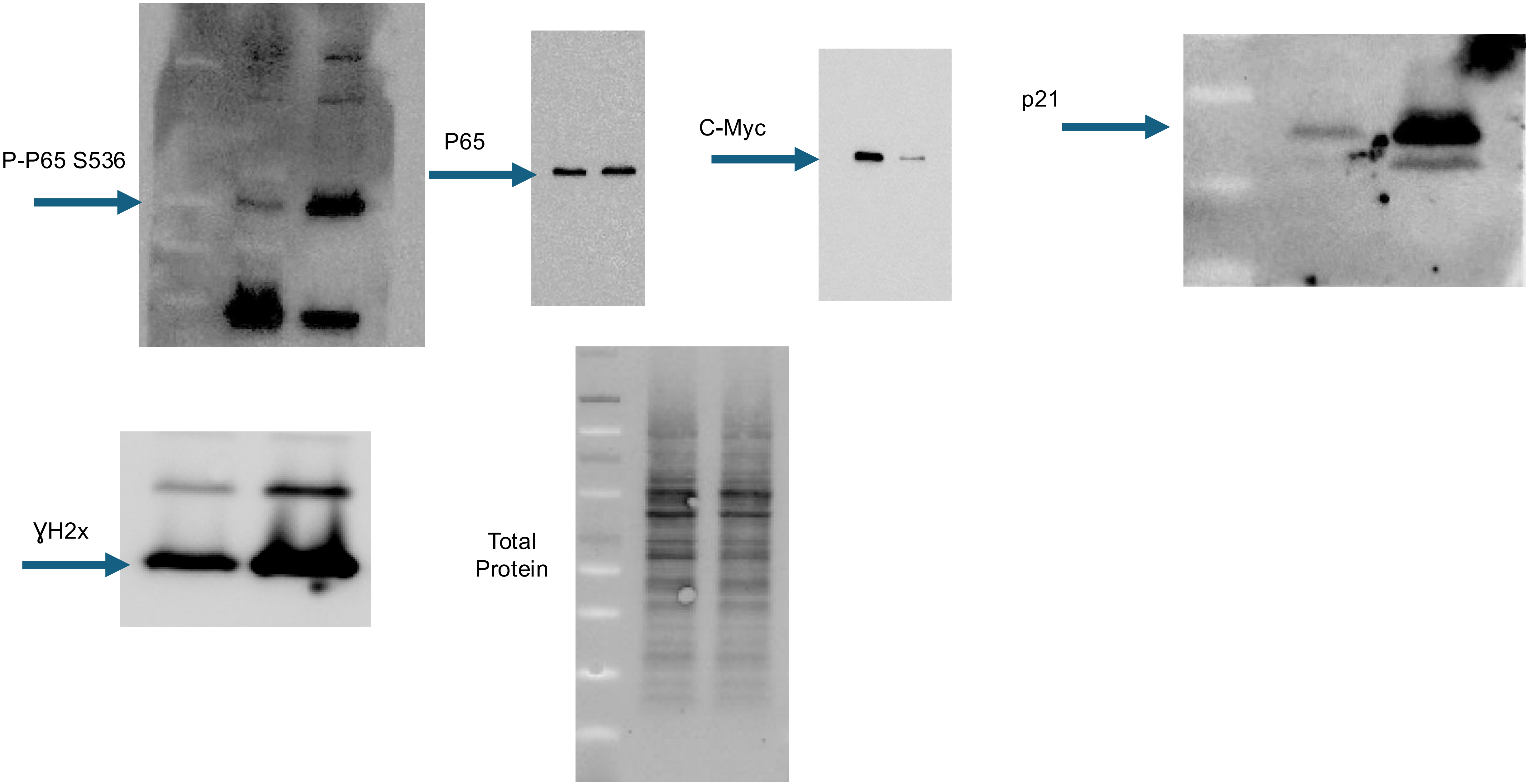

Full unedited gel for Figure 2I

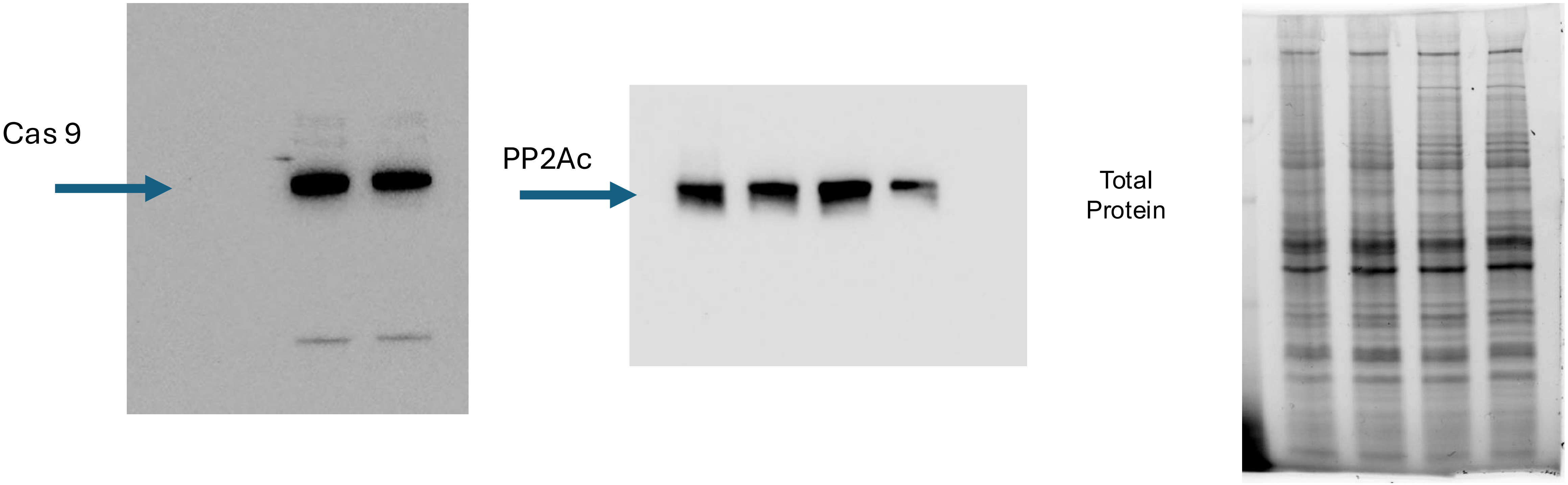

Full unedited gel for Figure 8E

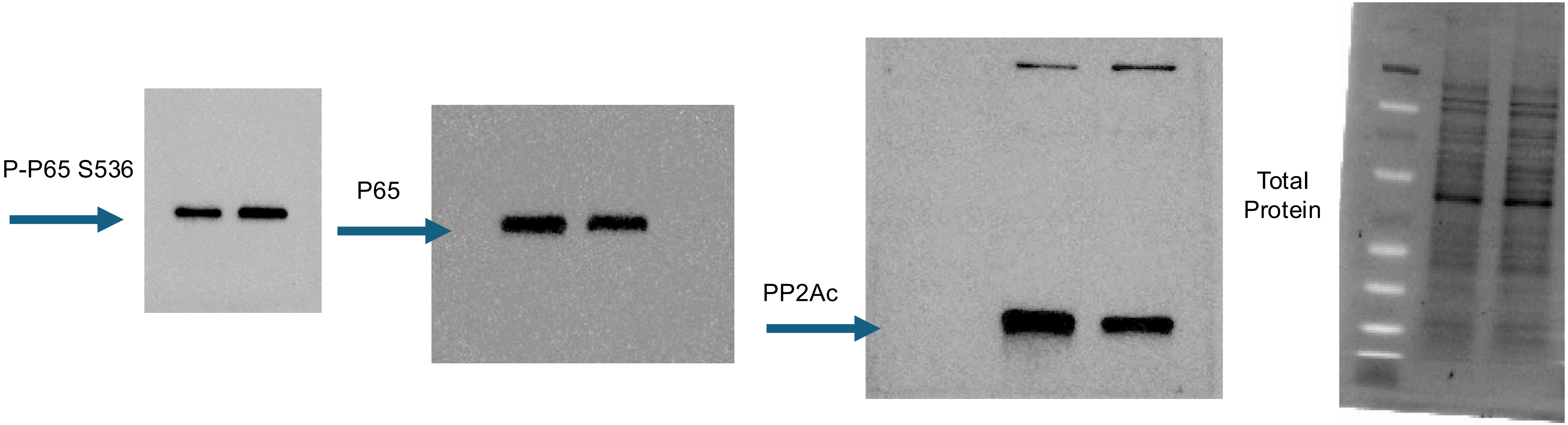

Full unedited gel for Figure S2A

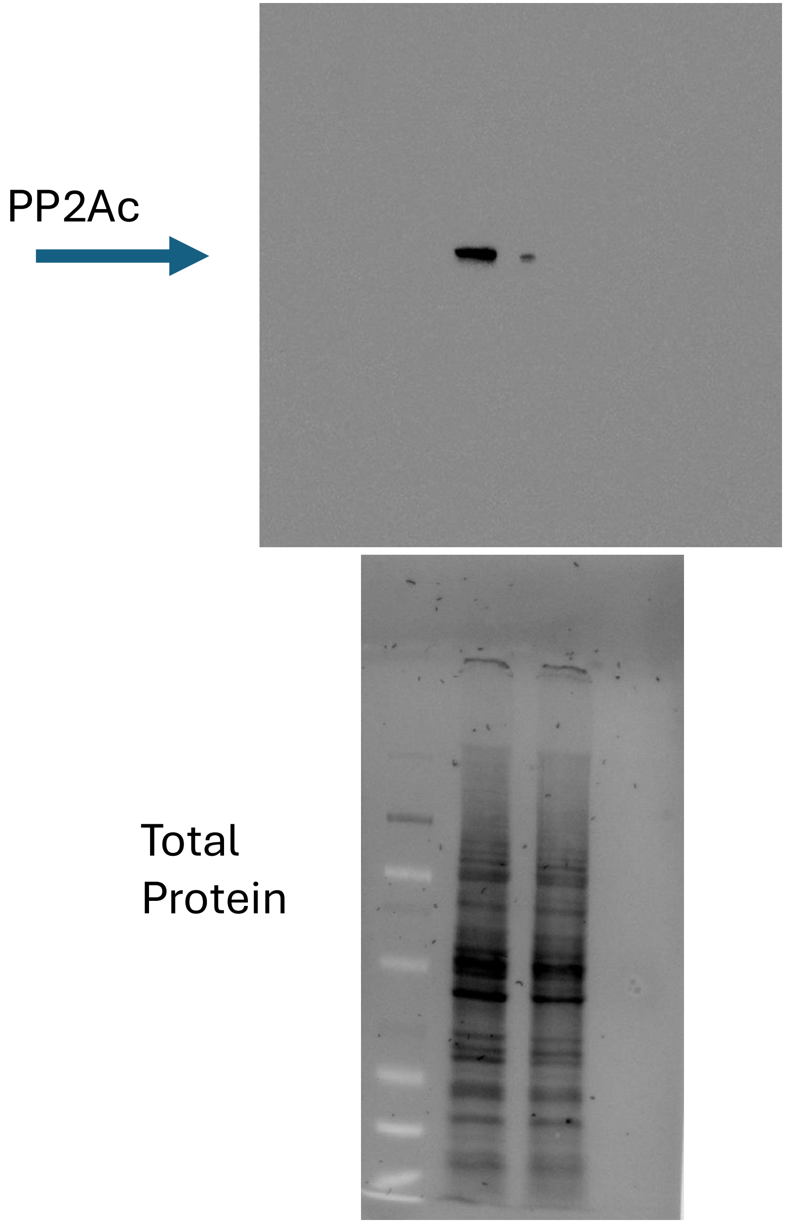

Full unedited gel for Figure S2B

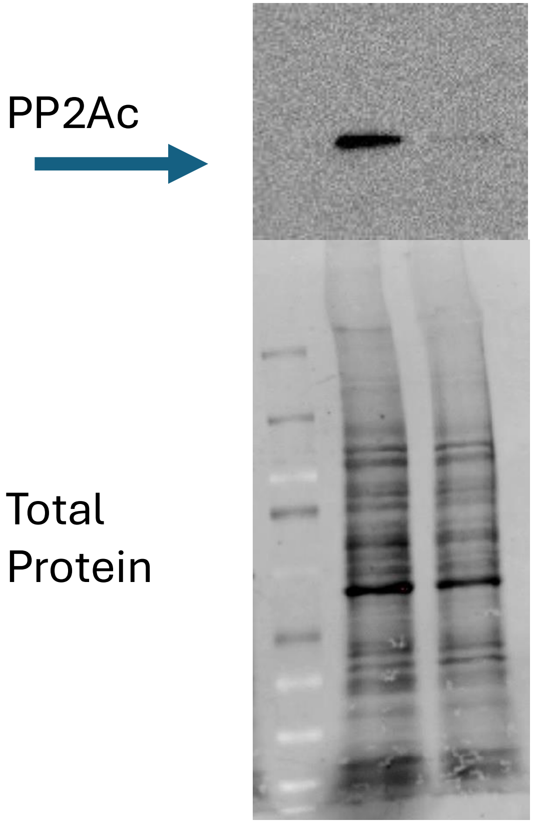

Indicates lanes shown in text

Full unedited gel for Figure S2C

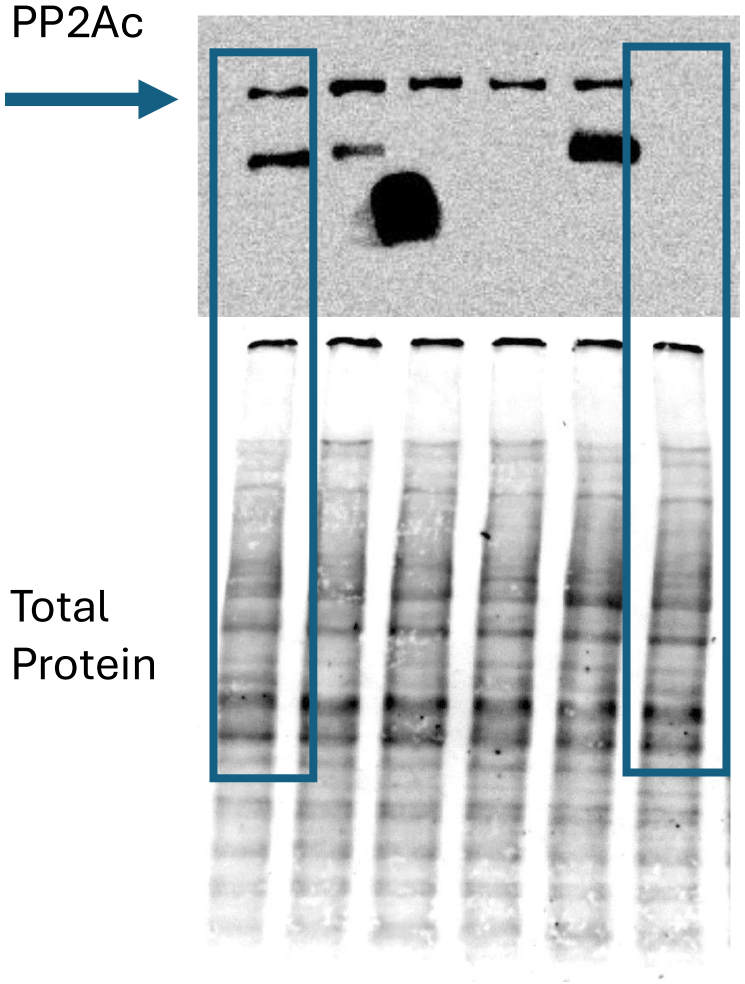

Full unedited gel for Figure S2D

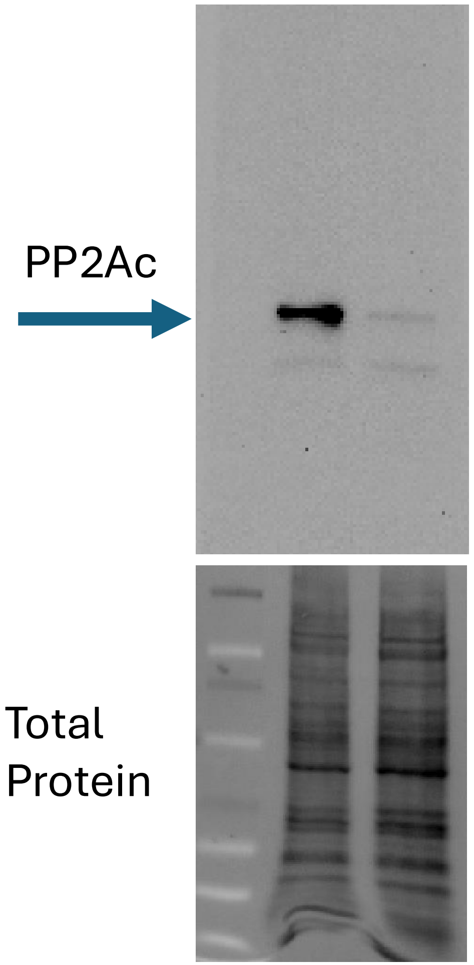

Indicates lanes shown in text

Full unedited gel for Figure S2E

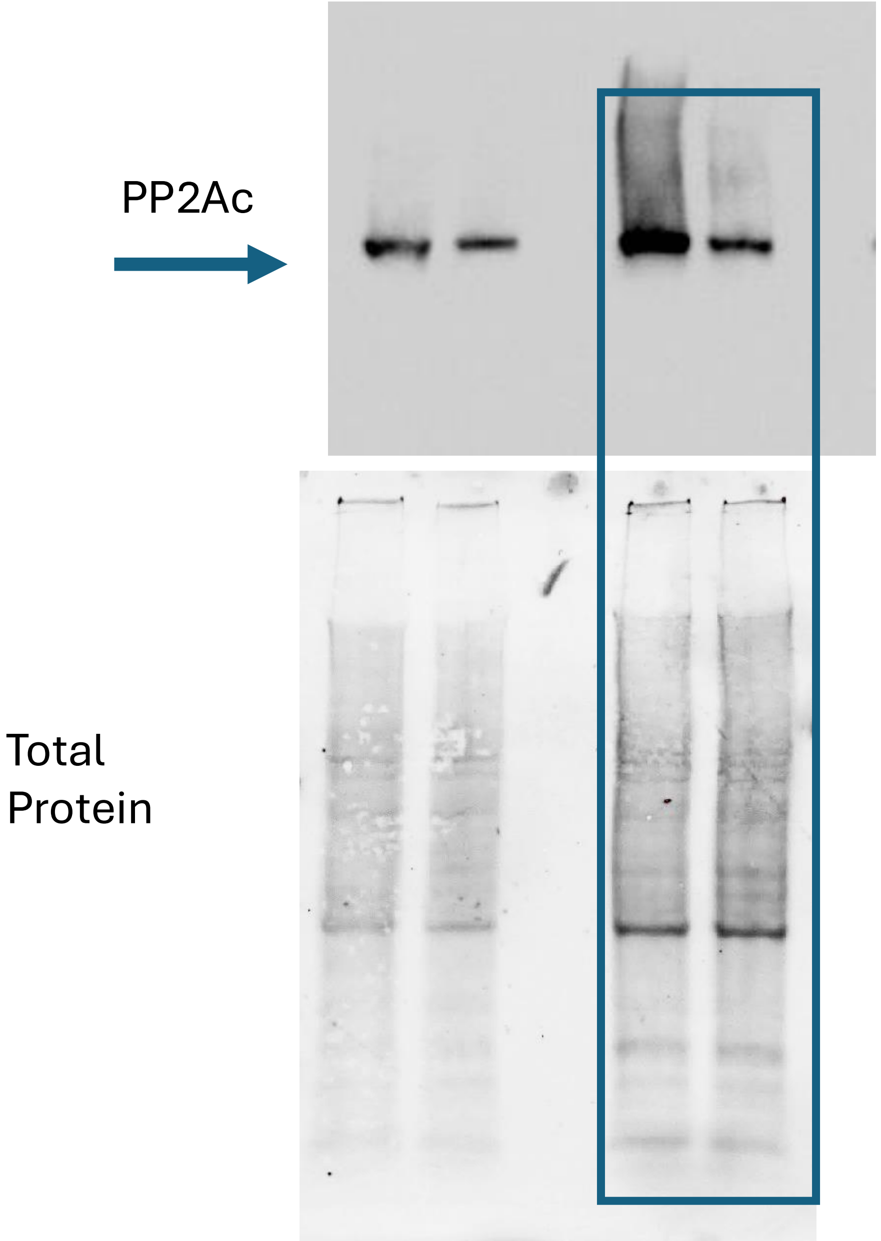

Full unedited gel for Figure S4

p-p65 (Ser536)

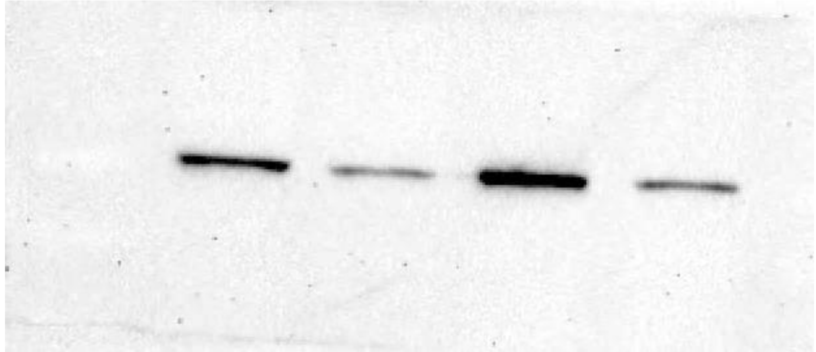

p65

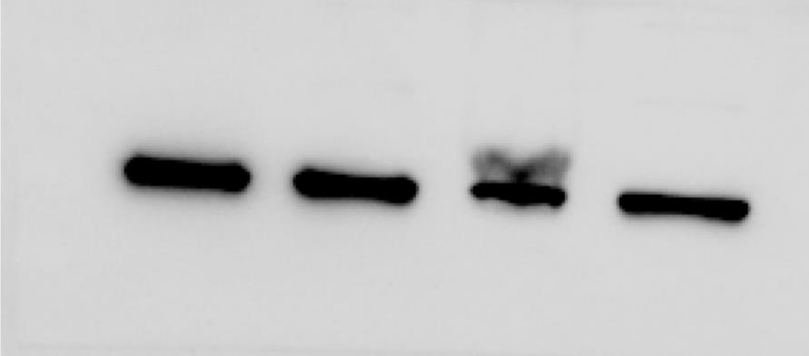

Total Protein

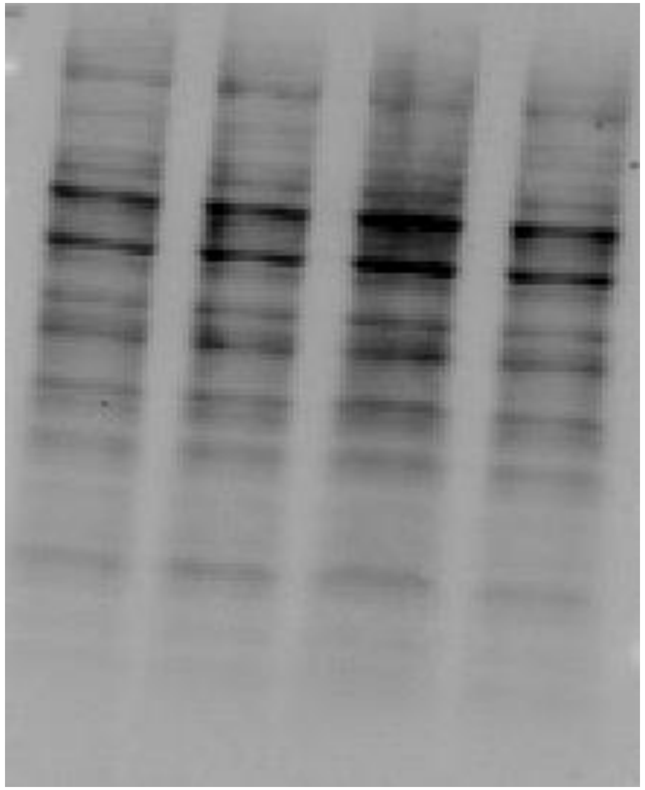

Full unedited gel for Figure S6C

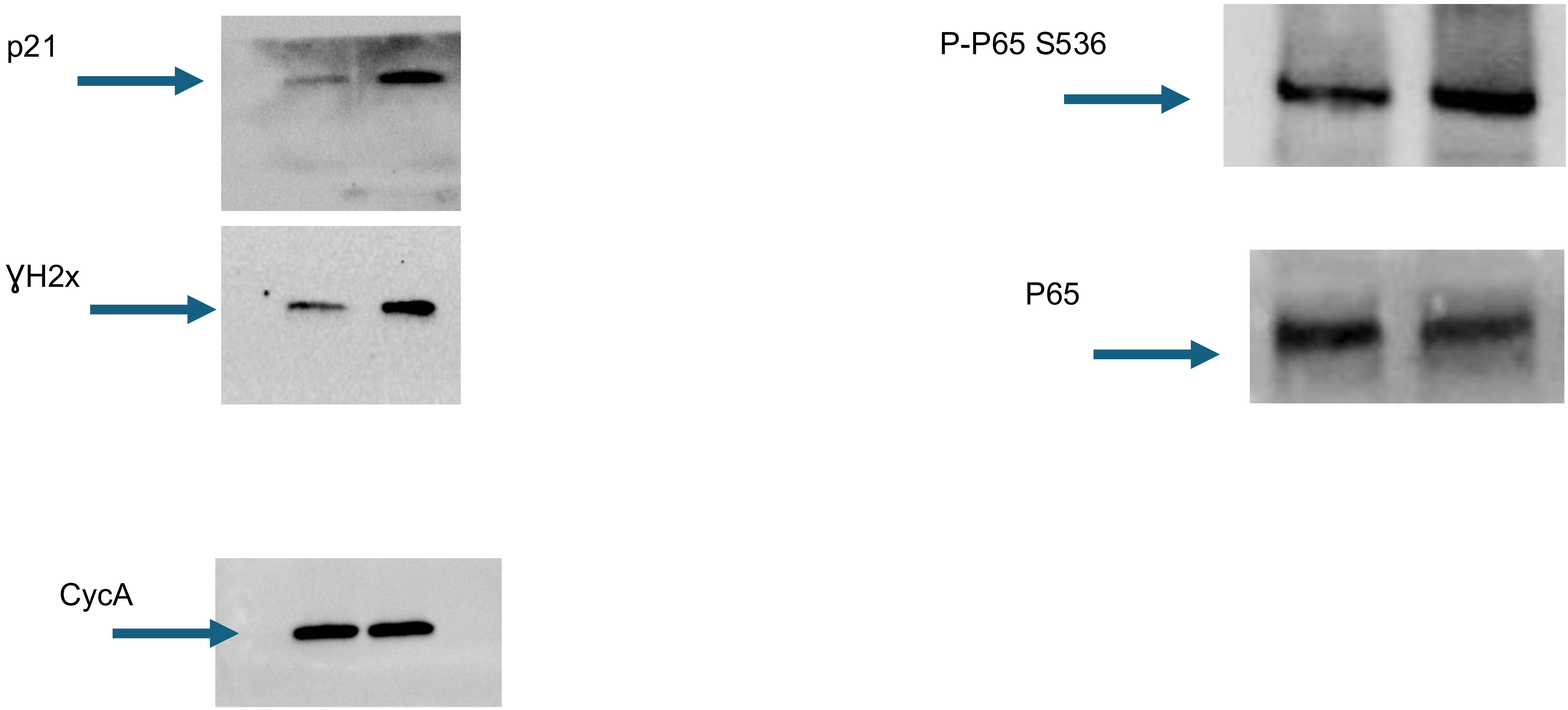

Supplement: Unedited blot and gel images [file jci-136-196753-s035.pdf]
